# Supplementary material for: Recurrent RNA edits in human preimplantation potentially enhance maternal mRNA clearance
Source: Commun Biol. 2022 Dec 21;5:1400. doi: 10.1038/s42003-022-04338-0 (PMC9772385; doi:10.1038/s42003-022-04338-0)
Supplement: Supplementary file 1 — Supplementary Information [file 42003_2022_4338_MOESM1_ESM.pdf]

Supplementary Information

Supplementary Note 1: Adaptation of a published pipeline for identification of the adenosine-to-inosine editome for human embryonic samples

We adapted a pipeline published by Ramaswami et al.[1] to identify the adenosine-to-inosine (A-to-I) RNA editome for human embryonic samples. Here we detail the adaptation steps. Also see Supplementary Figure 1 for a detailed pipeline flowchart.

Construction of splice-aware genomes

We used the human genome assembly hg38 and the University at California at Santa Cruz's knownGene GENCODE version 32[2] annotation to construct the splice-aware genomes. Because samples from the 20 datasets collected have different read lengths (Supplementary Data 1), we constructed a specific splice-aware genome for each read length. For a given paired-end or single-ended RNA sequencing (RNA-seq) sample with a read end length of n bp, the two transcript sequences n - 5 bp upstream and downstream of each junction site on a transcript were extracted (in the form of genomic plus strand sequences) and concatenated as the exonic sequence covering that junction site.

Alignment of reads to the splice-aware genome

We used Trim Galore! (version 0.6.6)[3] and then fastp[4] to automatically cut adapters and filter for reads with average quality scores >= 25 before mapping them to the splice-aware genome using Burrows-Wheeler alignment[5]. During this processing, we found that the sample GSM922196 (with Sequence Read Archive run accession SRR491011) contained invalid reads (i.e., reads whose sequence lengths were not equal to the quality lengths), which we removed. We then used Picard (`https://broadinstitute.github.io/picard/`) to merge runs from the same sample and removed the PCR du-

plicates. Finally, we mapped the coordinates of these alignments back onto the hg38 assembly, and kept only alignments with mapping quality scores >= 20 on chromosomes 1-22, X, or Y.

Variant calling with GATK

We used Genome Analysis Toolkit (GATK) version 3.6.0[6] to realign the reads at indels with IndelRealigner, and then recalibrated base quality scores using BaseRecalibrator with the dbSNP[7] vcf file (version 151) downloaded from `https://ftp.ncbi.nlm.nih.gov/snp/organisms/human_9606/VCF/00-common_all.vcf.gz` on August 26th, 2020. We called the variants using UnifiedGenotyper with the parameters "-stand_call_conf 0 -stand_emit_conf 0". All GATK commands were also equipped with the parameter "-U ALLOW_N_CIGAR_READS" when suggested by these commands.

Filter against unreliable variants

Steps (1-9) for first-round per-sample filtering (with relevant Perl script in the GitHub repository marked when available):

1. (`https://github.com/gao-lab/HERE/blob/master/scripts/S15_1__get_sample_RNA_editing_sites_v3/step07__apply_complex_filter/complex_filter_1/Convert_VCF.pl`) Starting from the GATK-called VCF file, we filtered variant sites ('sites' for short) for those on autosomes, chromosome X, or chromosome Y with the FILTER being "PASS" and at least one read covered.

2. (`https://github.com/gao-lab/HERE/blob/master/scripts/S15_1__get_sample_RNA_editing_sites_v3/step07__apply_complex_filter/complex_filter_1/ref_filter.pl`) We filtered for sites that had not been labeled as a dbSNP variant during GATK variant calling, or had been labeled as the cDNA

molecular type as annotated by UCSC Genome Browser dbSNP version 151 (http://genome.ucsc.edu/cgi-bin/hgTrackUi?db=hg38&g=snp151Flagged).

3. (https://github.com/gao-lab/HERE/blob/master/scripts/S15_1__get_sample_RNA_editing_sites_v3/step07__apply_complex_filter/complex_filter_1/threads_rmMismatch.pl and https://github.com/gao-lab/HERE/blob/master/scripts/S15_1__get_sample_RNA_editing_sites_v3/step07__apply_complex_filter/complex_filter_1/Remove_mismatch_first6bp.pl) For each site, we re-counted the number of mismatch reads on this sites based on all realigned reads (as produced by GATK IndelRealigner) whose mismatched base at this site was located outside the first 5-prime 6 bp of the read and had a base quality (as recorded in the realigned bam file) no less than 58. If no reads with mismatched bases were available for a given site after re-counting, this site was discarded. In addition, we discarded sites with two or more different types of mismatch bases (i.e., sites that are potential polymorphisms).

4. (https://github.com/gao-lab/HERE/blob/master/scripts/S15_1__get_sample_RNA_editing_sites_v3/step07__apply_complex_filter/complex_filter_1/Alu_filter.pl) We then separated the sites into those on and not on Alu elements, as annotated by the UCSC RepeatMasker track (http://genome.ucsc.edu/cgi-bin/hgTrackUi?db=hg38&g=rmsk); the resulting subsets were named "Alu subset" and "other subset", respectively. No sites were discarded in this step.

5. (https://github.com/gao-lab/HERE/blob/master/scripts/S15_1__get_sample_RNA_editing_sites_v3/step07__apply_complex_filter/complex_filter_1/FreqSimple.pl) We picked the "other subset" and filtered for sites that both (a) had a mismatch frequency > 0.1, and (b) did not overlap with any simple repeats described by the simple repeats annotated by the UCSC Genome

Browser RepeatMasker track.

6. (https://github.com/gao-lab/HERE/blob/master/scripts/S15_1__get_sample_RNA_editing_sites_v3/step07__apply_complex_filter/complex_filter_1/threads_rmSJandHomo.pl, https://github.com/gao-lab/HERE/blob/master/scripts/S15_1__get_sample_RNA_editing_sites_v3/step07__apply_complex_filter/complex_filter_1/RemoveHomoNucleotides.pl, and https://github.com/gao-lab/HERE/blob/master/scripts/S15_1__get_sample_RNA_editing_sites_v3/step07__apply_complex_filter/complex_filter_1/Filter_intron_near_splicejuncts.pl) We picked the output of Step (5), and filtered for sites that both (a) was not located within 4bp intronic regions of splicing sites defined by the UCSC Genome Browser knownGenes track (http://genome.ucsc.edu/cgi-bin/hgTrackUi?db=hg38&g=wgEncodeGencodeV32), and (b) was not located within a homopolymer >=5bp (i.e., a >=5bp sequence segment consisting of identical nucleotides).

7. (https://github.com/gao-lab/HERE/blob/master/scripts/S15_1__get_sample_RNA_editing_sites_v3/step07__apply_complex_filter/complex_filter_1/threads_BlatCandidates.pl and https://github.com/gao-lab/HERE/blob/master/scripts/S15_1__get_sample_RNA_editing_sites_v3/step07__apply_complex_filter/complex_filter_1/BLAT_candidates.pl) We picked the output of Step (6), and for each its site we (a) extracted all those realigned reads with the mismatched base for this site (as produced by GATK IndelRealigner), (b) aligned them to the entire genome with blat (with the following parameters: -stepSize=20 -repMatch=2253 -minScore=20 -minIdentity=0 -noHead) as suggested by Lo Giudice et al.[8], (c) identified for each read whether it can uniquely map to this site again (with its Blat score * 0.95 higher than the Blat score of the second best hit), and (d) kept this site only if the number of reads that can uniquely map to this site is no less than the number of reads that

can't. We then update the number of mismatches on this site with the number of reads that can uniquely map to this site.

8. (https://github.com/gao-lab/HERE/blob/master/scripts/S15_1__get_sample_RNA_editing_sites_v3/step07__apply_complex_filter/complex_filter_1/nonAlu_filter_new.pl) We picked the output of Step (7), and used UCSC Genome Browser RepeatMasker track to separate these sites into those that were on non-Alu repeat elements, and those that were not on repeat elements. No sites were discarded in this step.

9. We used "bcftools concat –allow-overlaps" to combine the sites called on Alu elements from Step (4), sites on non-Alu repeat elements from Step (8), and sites not on repeat elements from Step (8). No sites were discarded in this step.

Step (10)

We used "bcftools isec –collapse all" to identify all those genomic variants from the following worldwide population studies that overlapped with any of the sites discovered in any samples in Steps (1-9).

- the University of Washington Exome Sequencing Project (http://evs.gs.washington.edu/EVS/),
- the 1000Genomes Project[9] (http://ftp.1000genomes.ebi.ac.uk/vol1/ftp/data_collections/1000_genomes_project/release/20190312_biallelic_SNV_and_INDEL/),
- all version 2.1.1 exome and genome variants (using the liftover_hg38 versions) and all version 3.0 genome variants of the gnomAD project[10], and
- the National Center for Biotechnology Information's Allele Frequency Aggregator project (which involves the computation of allele frequencies for variants

in the database of Genotypes and Phenotypes across approved un-restricted studies)[11] (https://ftp.ncbi.nih.gov/snp/population_frequency/latest_release/freq.vcf.gz ; downloaded on November 11th, 2020).

We then used "bcftools isec –collapse all" to identify all those variant sites discovered in any samples in Steps (1-9) that overlapped with any of these genomic variants, and discarded these variant sites.

Step (11)

Starting with the variant sites kept in Step (10), we further kept those sites that met either of the following: (a) this site was located in an Alu element, and had >=10 reads covered, and had a >=10% mismatch frequency; (b) this site was not located in an Alu element, and had >=10 reads covered, and had a >=10% mismatch frequency, and had >=3 reads supporting the mismatch.

Step (12)

Starting with the variant sites kept in Step (11), we further kept those sites that met either of the following: (a) this site was located in an Alu element; (b) this site was not located in an Alu element, and was observed in at least two normal samples (or two abnormal samples) of the same stage (see Supplementary Data 2 for the details of each stage).

Step (13)

Starting with the variant sites kept in Step (11) and their SnpEff annotation (run with parameters "snpEff ann -lof" and the GENCODE v32 annotation), we kept those variants annotated with either the A>G (i.e., A-to-G) SnpEff event only, or both the A>G and the T>C (i.e., T-to-C) SnpEff event. The former can be regarded as strand-definite, while the latter can be regarded as strand-ambiguous.

We deliberately kept the latter type of variant, which appeared in at least two transcripts of different orientations (i.e., a variant reported by SnpEff to induce an A-to-G shift in one transcript and a thymine-to-cytosine shift in another), because a previously well-studied A-to-I RNA editing event found to recode tyrosine to cysteine in the *BLCAP* (bladder cancer-associated protein) gene[12], persist in human embryonic stem cells and multiple human tissues[13], and promote cell proliferation[14] is this type of variants.

Marking of unsequenced editing sites

Due to the inherent technical noise of RNA-seq that occurs with low cell content, including a high drop-out rate[15], the labeling of edits in unsequenced regions as nonexistent would be obviously biased. We thus marked all editing sites with zero read coverage in each sample as “unsequenced” rather than “nonexistent” or “undetected”; sites with at least one read covered were not altered in this step.

Supplementary Note 2: Validation of the reliability of the adapted pipeline for cells using paired DNA- and RNA-sequencing datasets

Ding et al.[16] suggested that the majority of mutations detected from single-cell RNA sequencing (scRNA-seq) datasets are likely to be [adenosine-to-inosine (A-to-I)] RNA editing events. Thus, the direct application of preexisting RNA editing identification pipelines, which are generally more stringent than genomic variant calling approaches[1] to these datasets, should yield a ratio of genomic variant-overlapping edits comparable to those obtained with bulk datasets.

Nevertheless, we validated our adapted pipeline using paired single-cell DNA-/RNA-seq datasets for the A375 cell line[17] (we did not use the dataset[18] used by Ding et al.[16] because it is not publicly available). For each A375 cell with both DNA and RNA sequenced, we downloaded the raw reads and applied our pipeline with the following modifications:

- We used Zachariadis et al.'s read preprocessing strategy[17] (`https://github.com/EngeLab/DNTRseq`).
- Whereas we applied all filters to RNA-seq data to obtain identified editing events, for DNA-Seq we stopped at the raw variant calling results generated by GATK and treated them as the ground truth for genomic variants.
- Due to the low sequencing depth of these samples, we adjusted the read coverage filter. Specifically, we filtered for Alu edits with at least two reads covered and an editing level of at least 0.1, and for non-Alu edits additionally with at least two reads with mismatches.

Comparison of the edits in each cell with its genomic variants revealed that all A375 cells with at least one edit identified had a zero ratio of genomic variant-overlapping

edits after, but not before, our filtering (Fig. 1d). This result supported the validity of our pipeline.

Supplementary Note 3: Analysis of Alu-editing index (AEI) across samples

We used the RNAEditingIndexer (https://github.com/a2iEditing/RNAEditingIndexer) as published by Roth et al. that starts from the RNA-seq read alignment (in BAM format) of a given sample and computes its AEI[19]. Because Roth et al. have demonstrated that their strand selection strategy (Fig. 1d and Methods in Roth et al.[19]) "has a high agreement between the [Alu-editing] indexes calculated both with and without taking into account the correct strand information (Fig. 2a, Supplementary Fig. 1, and Supplementary Notes 1)", for simplicity we assumed all datasets were unstranded.

The overall AEI is higher in 4-cells and earlier embryos and become much lower in 8-cells and later embryos (Supplementary Figure 7). Their correlations with *ADAR* expression are mostly either statistically significant but weak, or not statistically significant (Supplementary Figure 8), which is consistent with the observation that such correlation is rather weak in adult human tissues[19].

Supplementary Note 4: A preliminary case study on MBS-gaining REEs on a given gene

To investigate potential key targets of MBS-gaining REEs, we surveyed genes that have been shown to undergo maternal mRNA clearance, and examined whether they were targeted by any MBS-gaining REEs. We found that the *SUV39H2* gene, a known target of maternal mRNA clearance during human embryogenesis[20], was targeted by three REEs in oocytes (GV), one of which gains a new MBS, suggesting a potential REE-MBS-based mechanism of clearance regulation (Supplementary Figure 31). In support of this, a closer examination of their association with the targeted gene's expression level revealed a clear negative correlation (Supplementary Figure 32).

However, this MBS-gaining REE was classified as MBS-gaining only when we used the TargetScan prediction directly; when taking the intersection of predictions from TargetScan and miRanda, the TargetScan-predicted new MBS gained by this REE was not supported by miRanda. Therefore, we currently cannot determine whether this TargetScan-based discovery is merely a false positive or a true biological signal that is marked as a false negative by miRanda.

Supplementary Note 5: Analysis of REE on PAN2-PAN3, CCR4-NOT, and RNA exosome-mediated mRNA degradation pathways

We first examined whether the A-to-I edits could affect the key gene in each of these pathways. Specifically, we examined whether REEs target the following genes:

1. PAN2-PAN3[21]:
   (a) Core subunits: *PAN2*, *PAN3*
2. CCR4-NOT[22]:
   (a) Scaffold: *CNOT1*
   (b) Unknown but contributes to stabilization of the complex and RNA substrate recruitment: *CNOT2*, *CNOT10*, *CNOT11*
   (c) Interaction with ribosomes: *CNOT3*
   (d) Ubiquitin E3-ligase activity: *CNOT4*
   (e) Deadenylase: *CNOT6*, *CNOT6L*, *CNOT7*, *CNOT8*
   (f) Transcriptional cofactor: *CNOT9*
   (g) Multifunctional: *TNKS1BP1*
3. RNA exosome[23]:
   (a) Subunit of the 6-subunit ring of the 10-subunit core exosome: *EXOSC4*, *EXOSC5*, *EXOSC6*, *EXOSC7*, *EXOSC8*, *EXOSC9*
   (b) Subunit of the 3-subunit cap of the 10-subunit core exosome: *EXOSC1*, *EXOSC2*, *EXOSC3*
   (c) Ribonuclease/Catalytic subunit: *DIS3*

(d) Riboexonuclease subunit: *EXOSC10*

(e) Nuclear exosome cofactor: *TENT4B* (also known as *PAPD5*), *ZCCHC7*, *MTREX* (RNA helicase; also known as *MTR4*), *MPHOSPH6* (also known as *MPH6* and *MPP6*), *C1D*, *SETX*, *ZCCHC8*, *RBM7*

(f) Cytoplasmic exosome cofactor: *SKIV2L* (RNA helicase), *TTC37* (whose yeast ortholog is *Ski3*), *WDR61* (whose yeast ortholog is *Ski8*), *HBS1L* (isoform 3; whose yeast ortholog is *Ski7*)

We found that multiple REEs target *EXOSC6* in oocyte (GV), oocyte (MII), and zygote, and a single REE targets *CNOT6* in 2-cell (Supplementary Figure 34). While the function of *EXOSC6* has been rarely studied, the mouse ortholog of *EXOSC10* in the RNA exosome degradation pathway has been found to be required for the growth-to-maturation transition in oocytes[24] and eight-cell embryo/morula transition[25]; therefore, it is also possible that A-to-I editing on *EXOSC6* is a preferred regulation on the RNA exosome degradation pathway by oocytes (GV), oocytes (MII), and/or zygotes. On the other hand, the mouse ortholog of *CNOT6* gene has been found to regulate deadenylation of mRNAs in mouse oocyte growth and maturation[26], and the mouse CCR4-NOT complex itself is also found to be involved in regulating 2-cell-specific genes[27], suggesting the possibility that A-to-I editing on *CNOT6* might be preferred in the 2-cell stage as well.

Because the CCR4-NOT complex is known to target 3'-UTR of mRNAs with certain sequence motifs[28], we also examined whether REEs could lead to gain or loss of such motifs. Specifically, we examined the Pumilio-response element (PRE) UGUA-NAUW (where N is any nucleotide and W is either A or U)[29], and the AU-rich element (ARE) UUAUUUAUU[30]. In addition, we also examined the hnRNP A1 and A2/B1 binding site UAASUUAU (where S is either C or G) as discovered in[31]. We found that REEs in normal early stages did alter 6 motifs for 5 genes (Supplementary Figure 35), suggesting that A-to-I could indeed be able to affect (CCR4-NOT-based) RNA

degradation at the level of CCR4-NOT sequence motif.

Supplementary Note 6: A preliminary examination of the difference in the total number of REE-matching edits between abnormal and normal embryos

The (almost complete) loss of some REE-matching edits in embryos with uniparental disomy and those from elder mothers (Fig. 4) might be the extreme cases – the most affected REE-matching edits – of a wave of systematic loss of many (if not most) REE-matching edits in these embryos. Indeed, the number of REE-matching edits in abnormal embryos (or embryos from elder mothers) is smaller (though mostly statistically insignificantly) than that in normal embryos (or embryos from young mothers) in many but not all cases (Supplementary Figure 37). However, we noticed that the difference is indeed larger and clear in GSE95477. Therefore, we determined to examine whether there are certain subsets of protein-coding genes actively regulated by REEs that underwent statistically significant loss in abnormal embryos or embryos from elder mothers. To examine whether a subset of protein-coding genes actively regulated by REEs underwent statistically significant loss in abnormal embryos or embryos from elder mothers, we examined the average change in REEs per gene in targets of maternal mRNA clearance (using other maternal genes as controls) with the exclusion of samples that were potentially outliers (Supplementary Note 7). In GSE95477 samples (Supplementary Figure 38a), we observed statistically significantly fewer REE-matching edits per gene in targets of maternal mRNA clearance on average in oocytes (GV) from elder mothers than in those from young mothers (Supplementary Figure 38b), suggesting that REEs are favored in these targets at least at this developmental stage (see Supplementary Figure 41 for results from other datasets). These results raise the possibility that the loss of certain REEs is indicative of certain phenotypes in a manner that may be related to key embryonic processes such as maternal mRNA clearance. However, the sample size from which the conclusion is derived is too limited (no more

than 5 for each type of oocytes), and a further examination with a large sample size of each type of oocytes is needed to confirm whether this is a truly biological signal.

Supplementary Note 7: Outlier analysis with GSE95477

For analysis of distribution of REE-matching edits between targets of maternal mRNA clearance and other maternal genes (Supplementary Figure 38b), when taking all samples into account we observed a decline in the average number of recurrent embryonic edit (REE)-matching edits per gene in oocytes [germinal vesicle (GV) stage] from young to older mothers, but this difference was not statistically significant (Supplementary Figure 39). Further examination of these samples revealed that one from and older mother (GSM2514781) had a poor maturation rate relative to the moderate rates observed in other oocytes from older mothers (Supplementary Table 1 in[32]). We clustered all of the samples using a Manhattan distance based on all REE-matching edits of maternal genes only, and found that this sample appeared more like an oocyte from a young mother (Supplementary Figure 40). Thus, we excluded GSM2514781 (and GSM2514773, an oocyte from a young mother that clustered more closely with oocytes from older mothers) from the set of oocyte (GV) samples included in the analysis whose results are illustrated in Supplementary Figure 38b.

Supplementary Figures

Supplementary Figure 1

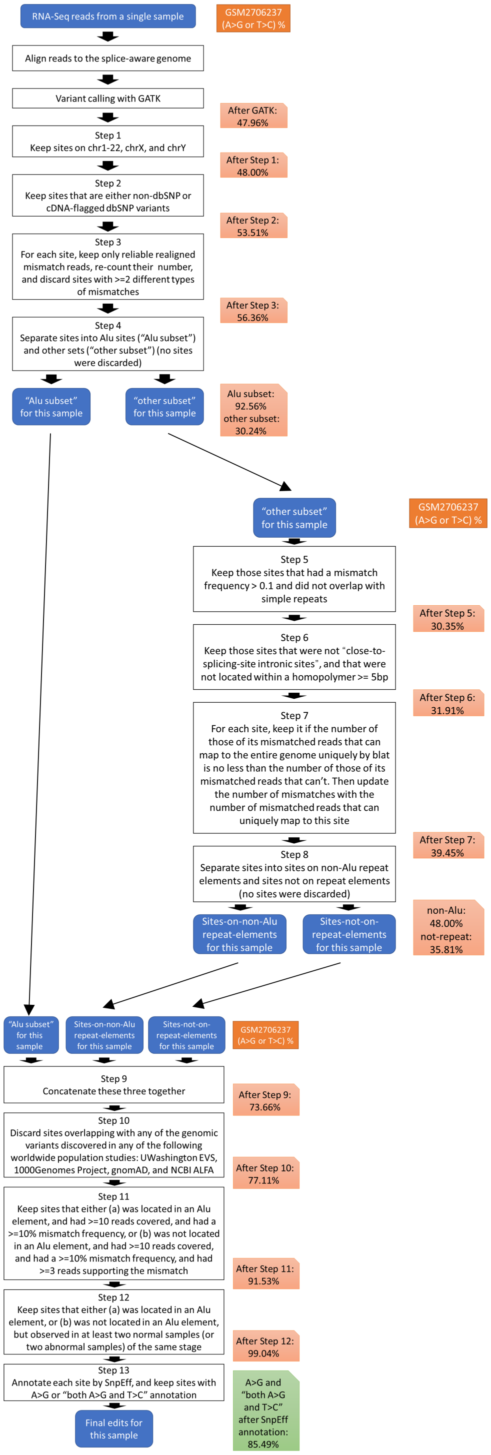

Supplementary Figure 1. The more detailed analysis pipeline flowchart. The per-filter A>G or T>C percentage (except for Step (13) where the A>G or "both A>G and T>C" percentage is used instead) of an example sample (GSM2706237) is listed.

Supplementary Figure 2

a

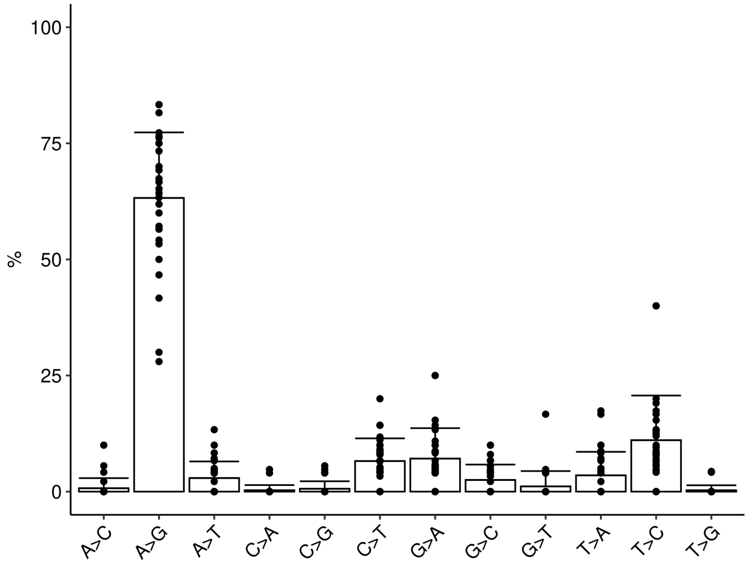

b

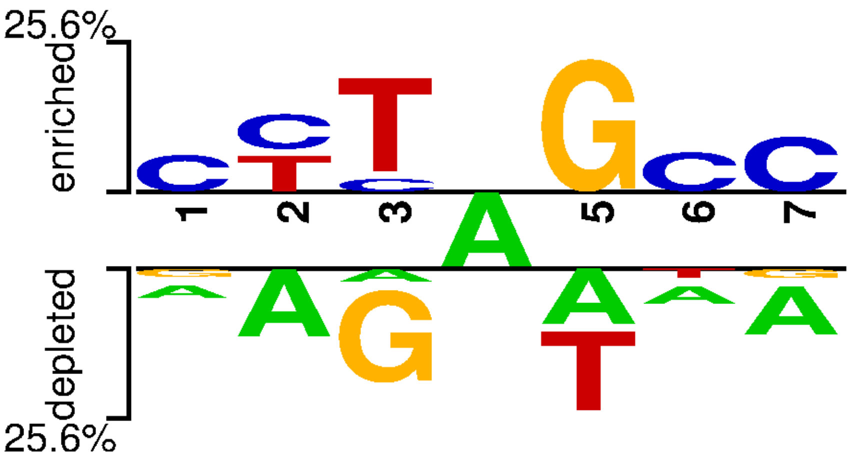

Supplementary Figure 2. Percentage of all possible 12 types of simple nucleotide changes and the triple motif of edits identified. (a), the all possible 12 types of simple nucleotide changes. (b), the triple motif of edits identified. In (a), bar height

and error bars display the mean and standard deviation, respectively, of percentage across the samples. Only samples with at least 10 edits identified were considered in this plot.

Supplementary Figure 3

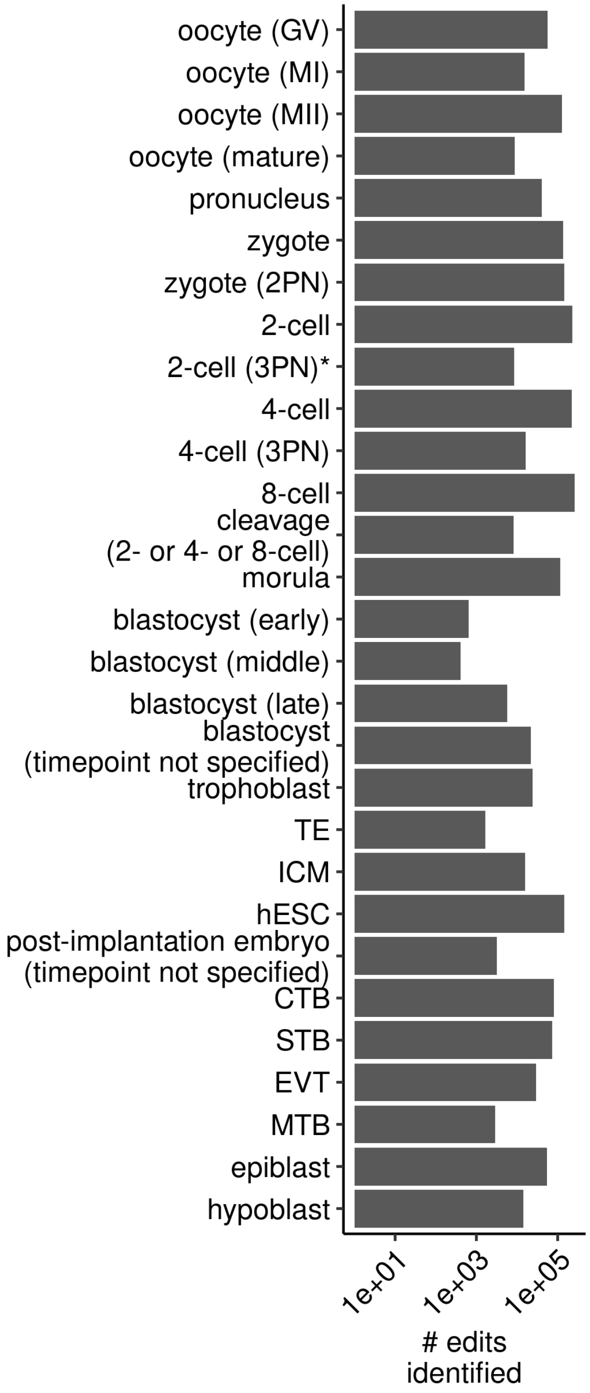

Supplementary Figure 3. Counts of the union of edits identified across all samples from each stage. Stages labeled with * have only one sample. GV, germinal vesicle. MI, metaphase of first meiosis. MII, metaphase of second meiosis. PN, pronuclear. TE, trophectoderm. ICM, inner cell mass. hESC, human embryonic stem cell. CTB, cytotrophoblast. STB, syncytiotrophoblast. EVT, extravillous trophoblast. MTB, migratory trophoblast.

Supplementary Figure 4

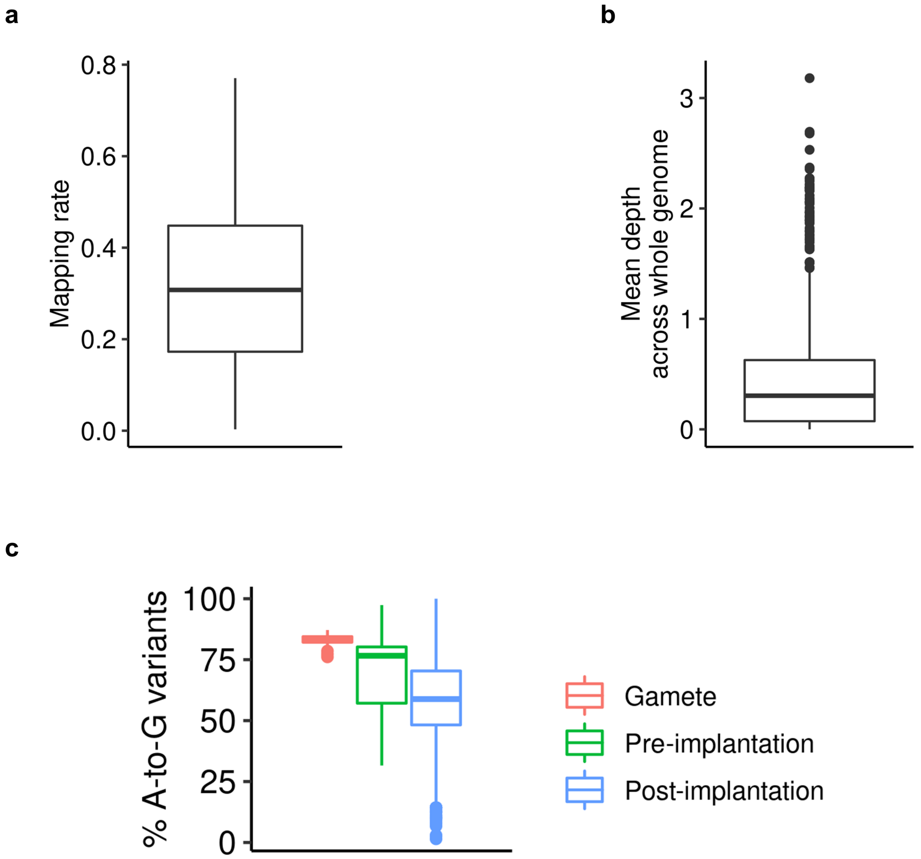

Supplementary Figure 4. The distribution plot of mapping and variant calling statistics for the samples analyzed. (a), distribution for the mapping rates (defined as the ratio of "the number of reads kept after GATK recalibration" to "the total number of trimmed reads prior to BWA mapping"). (b), distribution for the sequencing depths (defined as the samtools coverage-reported mean depth across the whole genome). (c), distribution for the A-to-G proportions across all 12 nucleotide changes. Symbols in boxplots follow the definition by "geom_boxplot" of the R package "ggplot2"[33] : the inner thick line indicates the median; the lower and upper boundaries (or hinges) of the box indicate the first and third quartiles (i.e., 25% and 75% quantiles), respectively; the upper whisker extends from the hinge to the largest value no further than

1.5 * inter-quartile range (the third quartile minus the first quartile), the lower whisker extends from the hinge to the smallest value at most 1.5 * inter-quartile range of the hinge, and data beyond the end of the whiskers are the outlier points, which are plotted individually.

Supplementary Figure 5

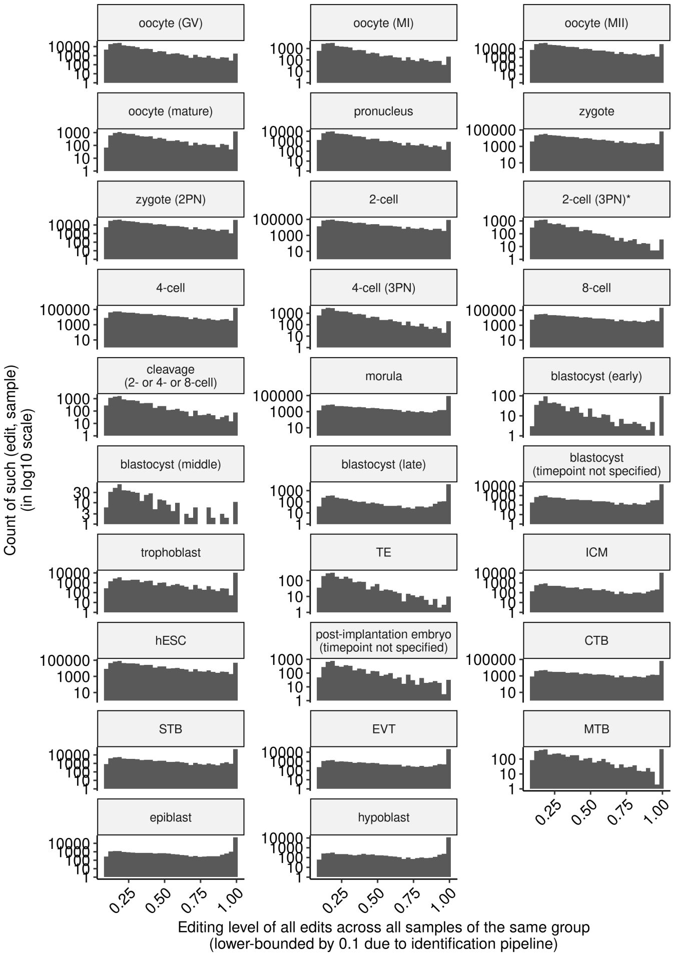

Supplementary Figure 5. Distribution of editing level in each sample group (with all edits shown and in log10-scale). Note that although we have used a very stringent pipeline (see Methods), we still found a number of editing sites with an editing level of 1. These editing sites might not be real A-to-I edits and should be examined with caution. Here all edits were shown, and the y-axis was plotted in log10-scale.

Supplementary Figure 6

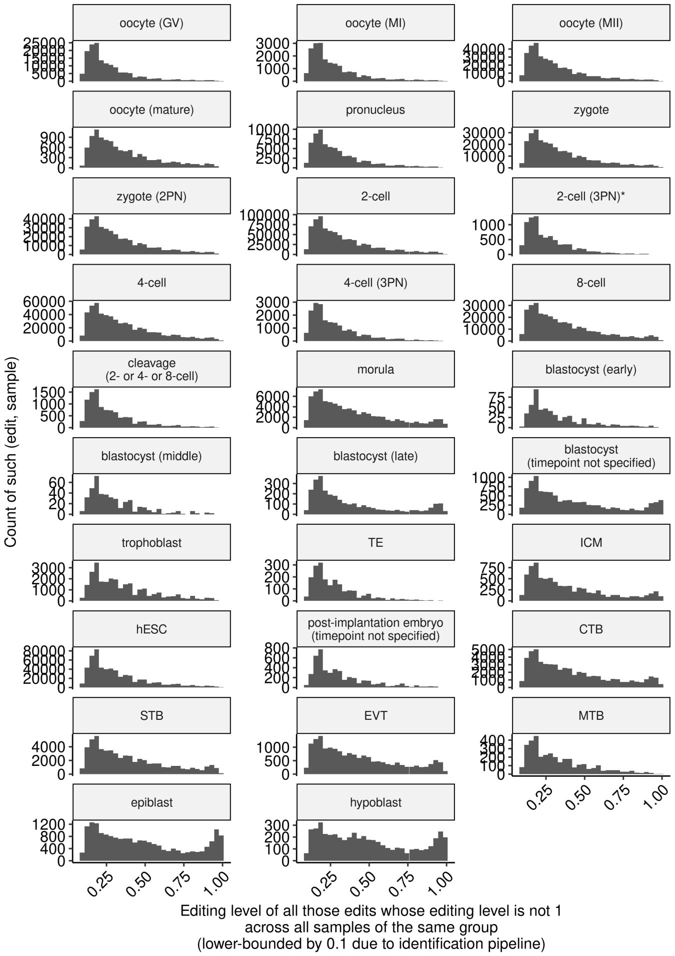

Supplementary Figure 6. Distribution of editing level in each sample group (where only edits with editing level not being 1 were shown). This figure is the same as Supplementary Figure 5 except that only edits with editing level not being 1 were plotted, and the y-axis was plotted as-is.

Supplementary Figure 7

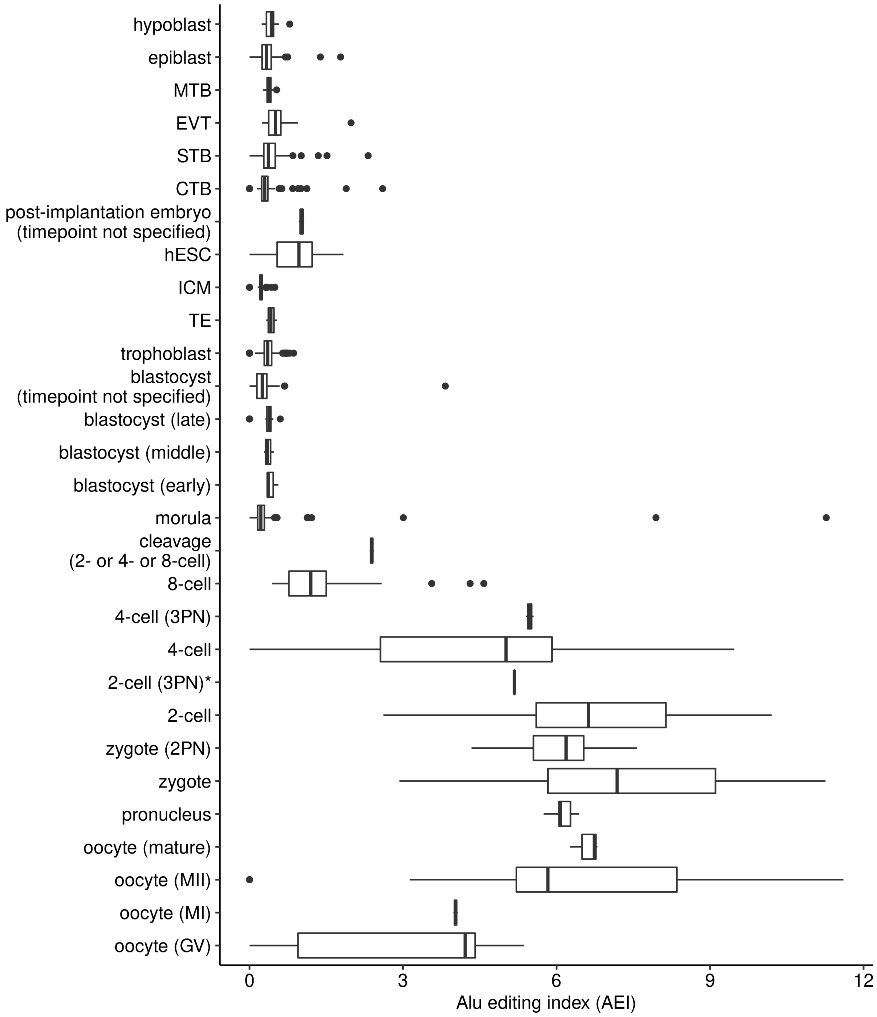

Supplementary Figure 7. Distribution of AEI across different stages of embryonic samples. Symbols in boxplots follow the definition by "geom_boxplot" of the R package "ggplot2"[33] : the inner thick line indicates the median; the lower and upper boundaries (or hinges) of the box indicate the first and third quartiles (i.e., 25% and 75% quantiles), respectively; the upper whisker extends from the hinge to the largest value no further than 1.5 * inter-quartile range (the third quartile minus the first

quartile), the lower whisker extends from the hinge to the smallest value at most 1.5 * inter-quartile range of the hinge, and data beyond the end of the whiskers are the outlier points, which are plotted individually.

Supplementary Figure 8

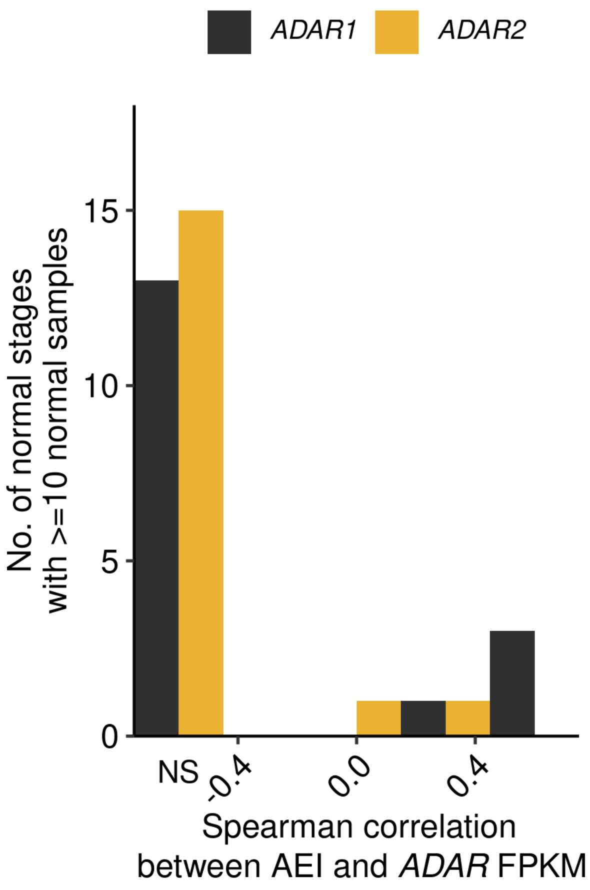

Supplementary Figure 8. Histogram of Spearman's correlation coefficients between AEI and *ADAR* FPKM for each normal stage with >=10 normal samples. All those that have a Benjamini-Hochberg-adjusted p-value for the Spearman correlation coefficient test (with the alternative hypothesis being that the correlation coefficient is not equal to 0) that is no less than 0.05 were considered not significant (NS). See Supplementary Data 33 for description of sample size and the 95% confidence interval for each of these tests.

Supplementary Figure 9

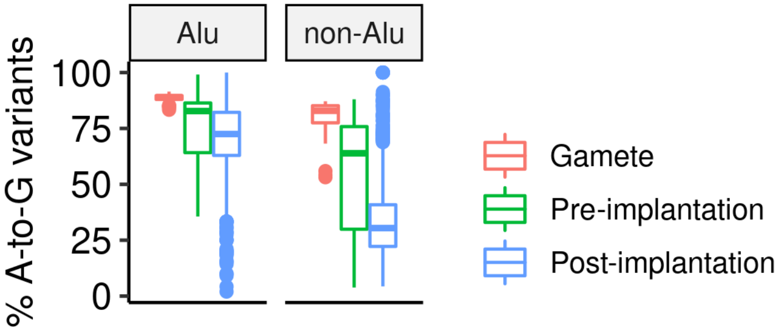

Supplementary Figure 9. Distribution of percentage of A-to-G variants for Alu and non-Alu sites separately. The proportion is defined as "the union of strand-definite A-to-G variants and strand-ambiguous A-to-G/T-to-C variants" (see Step (13) of Supplementary Note 1) to all variants. Symbols in boxplots follow the definition by "geom_boxplot" of the R package "ggplot2"[33] : the inner thick line indicates the median; the lower and upper boundaries (or hinges) of the box indicate the first and third quartiles (i.e., 25% and 75% quantiles), respectively; the upper whisker extends from the hinge to the largest value no further than 1.5 * inter-quartile range (the third quartile minus the first quartile), the lower whisker extends from the hinge to the smallest value at most 1.5 * inter-quartile range of the hinge, and data beyond the end of the whiskers are the outlier points, which are plotted individually.

Supplementary Figure 10

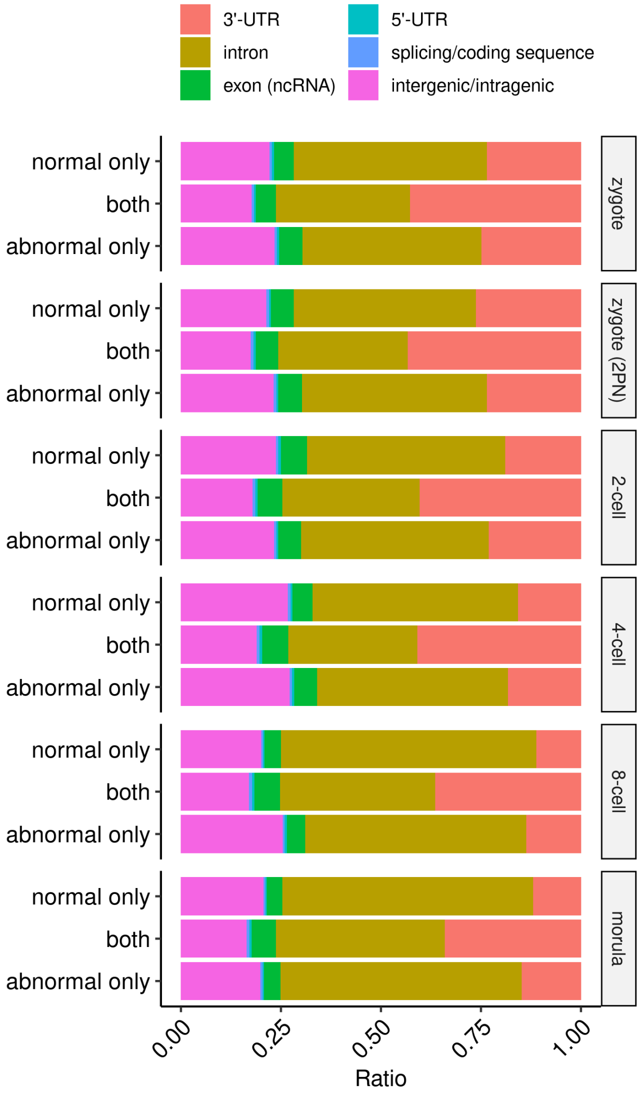

Supplementary Figure 10. Overall genomic distribution of edits from normal and/or abnormal samples per stage. Only stages with both types of samples were shown.

Supplementary Figure 11

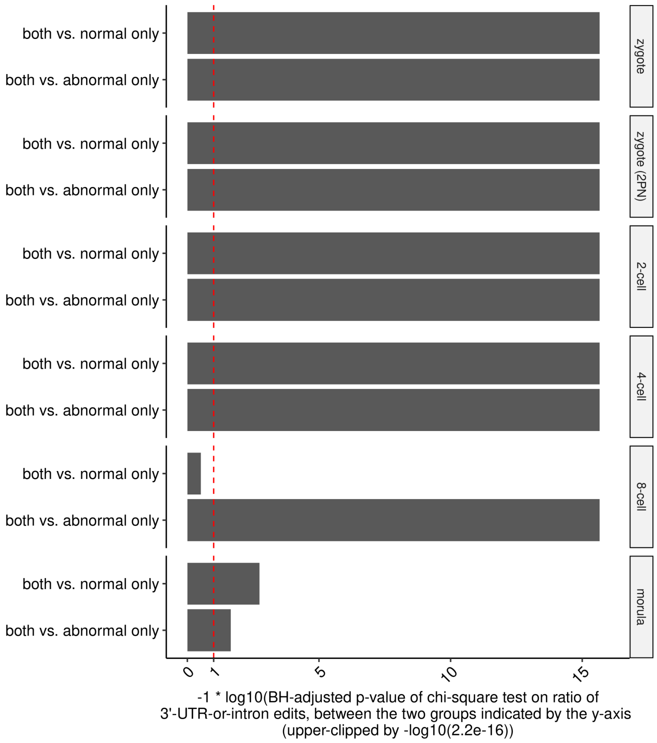

Supplementary Figure 11. Statistical significance in the deviation of 3'-UTR-or-intron edit ratio of the normal-abnormal overlap edits from that of the normal-/abnormal-unique edits per stage. While their overall distribution (see Supplementary Figure 10) displayed a statistical significance in the deviation of 3'-UTR-or-intron edit ratio of the overlap edits from that of the unique edits, as shown here, such deviation might partially arise from the fact that they were also identified with a higher sequence coverage (see Supplementary Figure 12). In addition, the drop of statistical significance in 8-cells and morula stages might be a consequence of multiple factors, including but not limited to the loss of a stable editing pattern indicated by REE upon entry into the 8-cell stage (Fig. 2b), the end of maternal mRNA clearance[34], and the wave of zygotic genomic activation[35] in the 8-cell stage. Only stages with both types of samples were shown. All p-values were from the chi-square test (with the null hypothe-

sis being that whether an edit is a 3’-UTR-or-intron edit is independent of whether this edit is shared by both or normal-/abnormal-only) and Benjamini-Hochberg-adjusted. See Supplementary Data 28 (the source data behind Supplementary Figures 10 and 11) for the number of edits in each group in each chi-square test.

Supplementary Figure 12

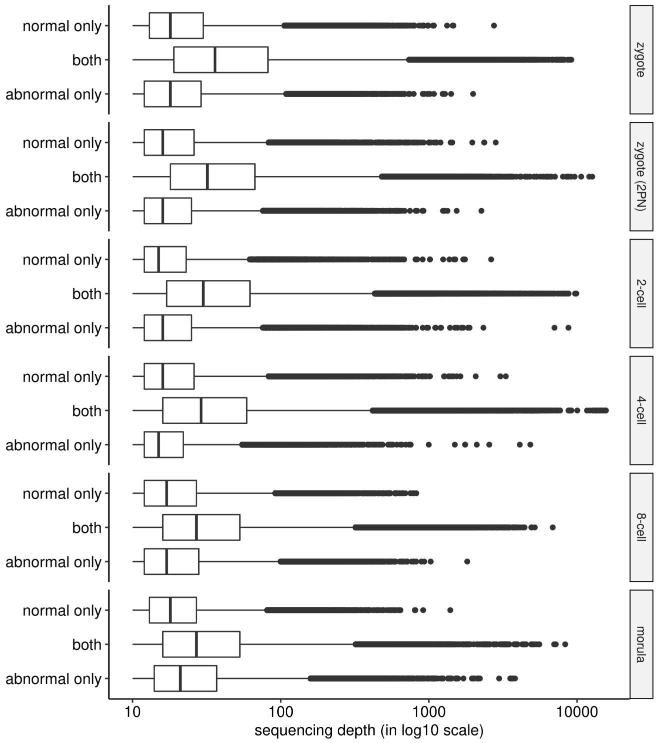

Supplementary Figure 12. Distribution of per-sample sequencing depth of the normal-abnormal overlap edits from that of the normal-/abnormal-unique edits per stage. See Supplementary Figure 11 for more details. Only stages with both types of samples were shown. Symbols in boxplots follow the definition by "geom_boxplot" of the R package "ggplot2"[33] : the inner thick line indicates the median; the lower and upper boundaries (or hinges) of the box indicate the first and third quartiles (i.e., 25% and 75% quantiles), respectively; the upper whisker extends from the hinge to the largest value no further than 1.5 * inter-quartile range (the third quartile minus the first quartile), the lower whisker extends from the hinge to the smallest value at most 1.5 * inter-quartile range of the hinge, and data beyond the end of the whiskers are the outlier points, which are plotted individually.

Supplementary Figure 13

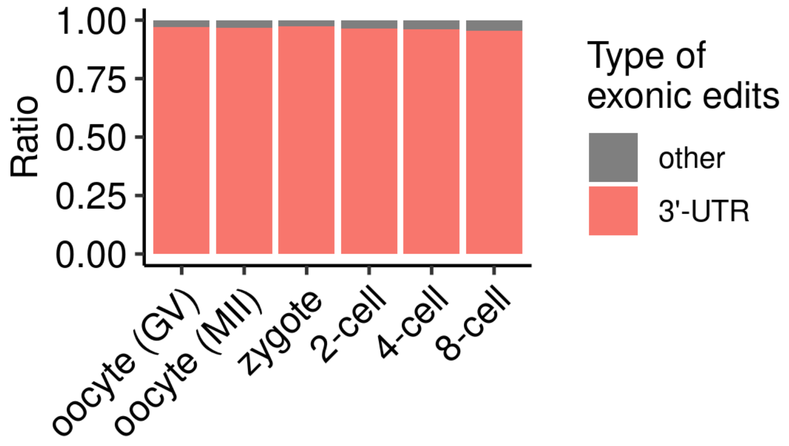

Supplementary Figure 13. Percentage of 3'-UTR edits among all edits observed in the early stages of embryogenesis.

Supplementary Figure 14

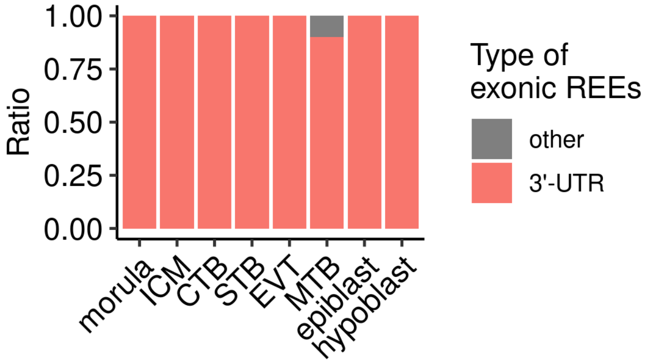

Supplementary Figure 14. Percentage of 3’-UTR REEs in the late stages of embryogenesis.

Supplementary Figure 15

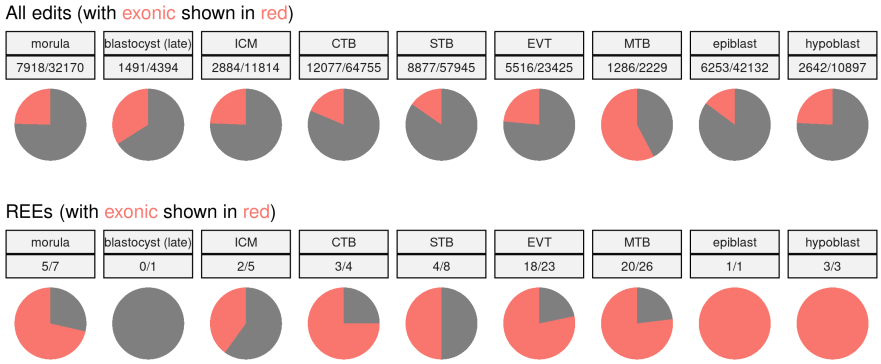

Supplementary Figure 15. Percentage of exonic edits and exonic REEs (shown in red) in the late stages of embryogenesis. Each stage label is appended with an additional label describing the number of exonic edits (or REEs) / the number of total edits (or REEs).

Supplementary Figure 16

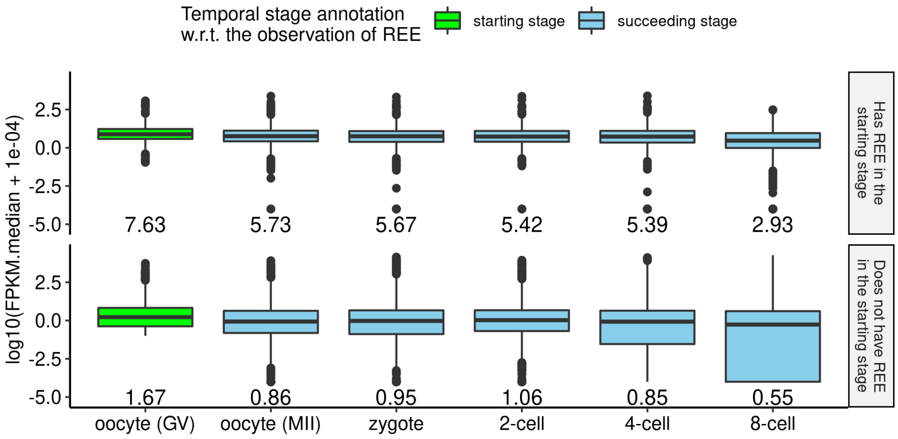

Supplementary Figure 16. The developmental expression pattern of genes with and without REEs, with the starting stage being oocyte (GV). For each of Supplementary Figure 16, Supplementary Figure 17, Supplementary Figure 18, Supplementary Figure 19, and Supplementary Figure 20, the starting stage is the stage whose observation of REE on each gene was used to split the genes into “Has REE in the starting stage” and “Does not have REE in the starting stage”, and the “subsequent stage” is the stage that is after the starting stage along the development timeline. In addition, for each subplot only genes with FPKM > 0.1 at the starting stage was considered. The number shown below each boxplot is the median of all FPKM medians (without the transformation of $\log_{10}(\text{FPKM median} + 1 \times 10^{-4})$) for that boxplot. Here, the starting stage is oocyte (GV). Symbols in boxplots follow the definition by “geom_boxplot” of the R package “ggplot2”[33] : the inner thick line indicates the median; the lower and upper boundaries (or hinges) of the box indicate the first and third quartiles (i.e., 25% and 75% quantiles), respectively; the upper whisker extends from the hinge to the largest value no further than 1.5 * inter-quartile range (the third quartile minus the first quartile), the lower whisker extends from the hinge to the smallest value at most 1.5 * inter-quartile range of the hinge, and data beyond the end of the whiskers

are the outlier points, which are plotted individually.

Supplementary Figure 17

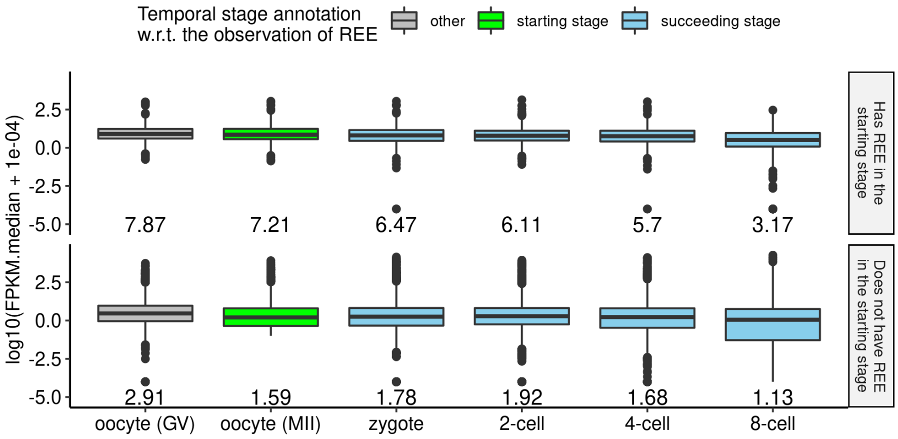

Supplementary Figure 17. The developmental expression pattern of genes with and without REEs, with the starting stage being oocyte (MII). See Supplementary Figure 16 for more details. Symbols in boxplots follow the definition by "geom_boxplot" of the R package "ggplot2"[33] : the inner thick line indicates the median; the lower and upper boundaries (or hinges) of the box indicate the first and third quartiles (i.e., 25% and 75% quantiles), respectively; the upper whisker extends from the hinge to the largest value no further than 1.5 * inter-quartile range (the third quartile minus the first quartile), the lower whisker extends from the hinge to the smallest value at most 1.5 * inter-quartile range of the hinge, and data beyond the end of the whiskers are the outlier points, which are plotted individually.

Supplementary Figure 18

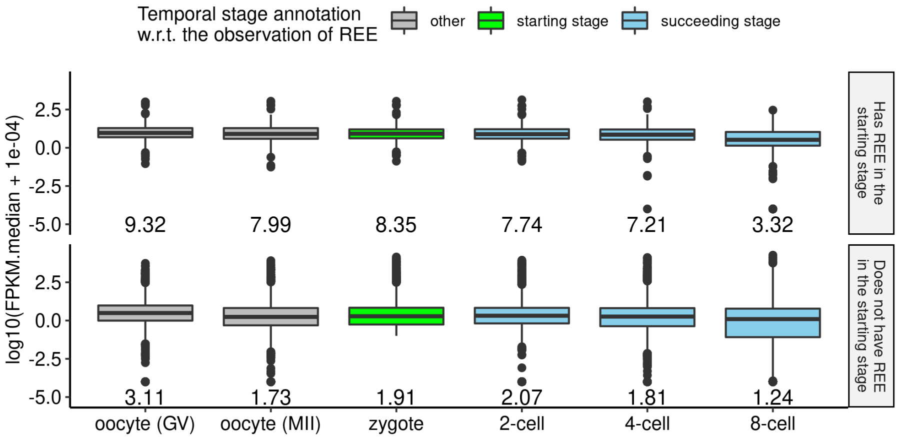

Supplementary Figure 18. The developmental expression pattern of genes with and without REEs, with the starting stage being zygote. See Supplementary Figure 16 for more details. Symbols in boxplots follow the definition by "geom_boxplot" of the R package "ggplot2"[33] : the inner thick line indicates the median; the lower and upper boundaries (or hinges) of the box indicate the first and third quartiles (i.e., 25% and 75% quantiles), respectively; the upper whisker extends from the hinge to the largest value no further than 1.5 * inter-quartile range (the third quartile minus the first quartile), the lower whisker extends from the hinge to the smallest value at most 1.5 * inter-quartile range of the hinge, and data beyond the end of the whiskers are the outlier points, which are plotted individually.

Supplementary Figure 19

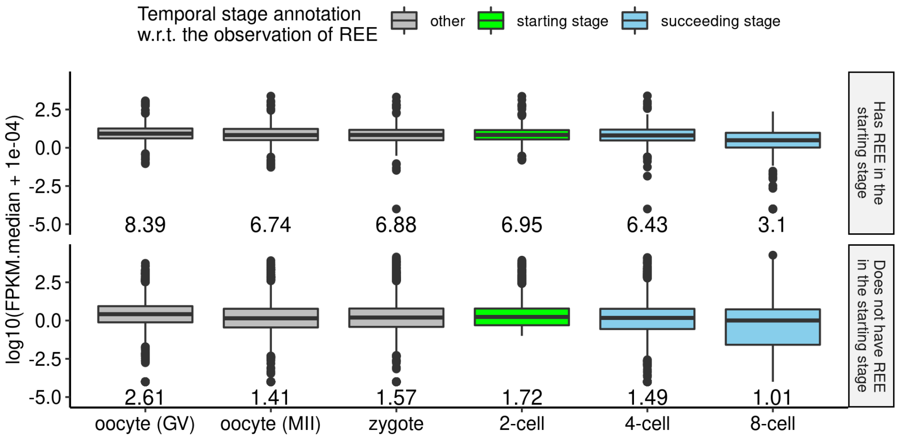

Supplementary Figure 19. The developmental expression pattern of genes with and without REEs, with the starting stage being 2-cell. See Supplementary Figure 16 for more details. Symbols in boxplots follow the definition by "geom_boxplot" of the R package "ggplot2"[33] : the inner thick line indicates the median; the lower and upper boundaries (or hinges) of the box indicate the first and third quartiles (i.e., 25% and 75% quantiles), respectively; the upper whisker extends from the hinge to the largest value no further than 1.5 * inter-quartile range (the third quartile minus the first quartile), the lower whisker extends from the hinge to the smallest value at most 1.5 * inter-quartile range of the hinge, and data beyond the end of the whiskers are the outlier points, which are plotted individually.

Supplementary Figure 20

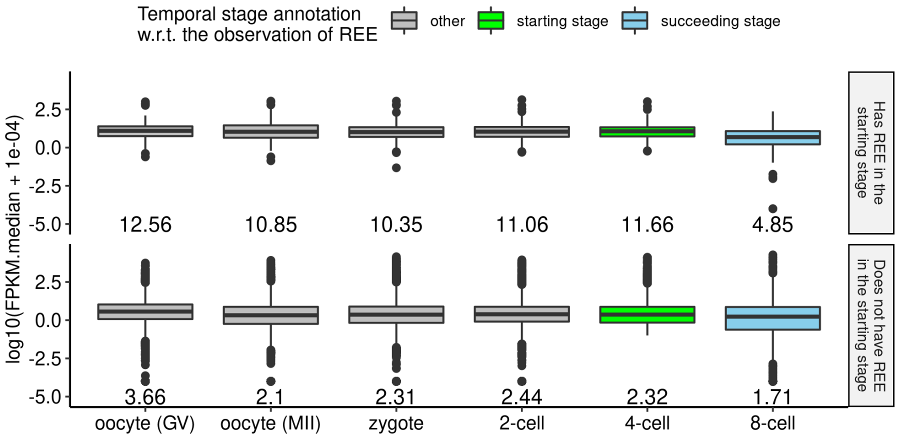

Supplementary Figure 20. The developmental expression pattern of genes with and without REEs, with the starting stage being 4-cell. See Supplementary Figure 16 for more details. Symbols in boxplots follow the definition by "geom_boxplot" of the R package "ggplot2"[33] : the inner thick line indicates the median; the lower and upper boundaries (or hinges) of the box indicate the first and third quartiles (i.e., 25% and 75% quantiles), respectively; the upper whisker extends from the hinge to the largest value no further than 1.5 * inter-quartile range (the third quartile minus the first quartile), the lower whisker extends from the hinge to the smallest value at most 1.5 * inter-quartile range of the hinge, and data beyond the end of the whiskers are the outlier points, which are plotted individually.

Supplementary Figure 21

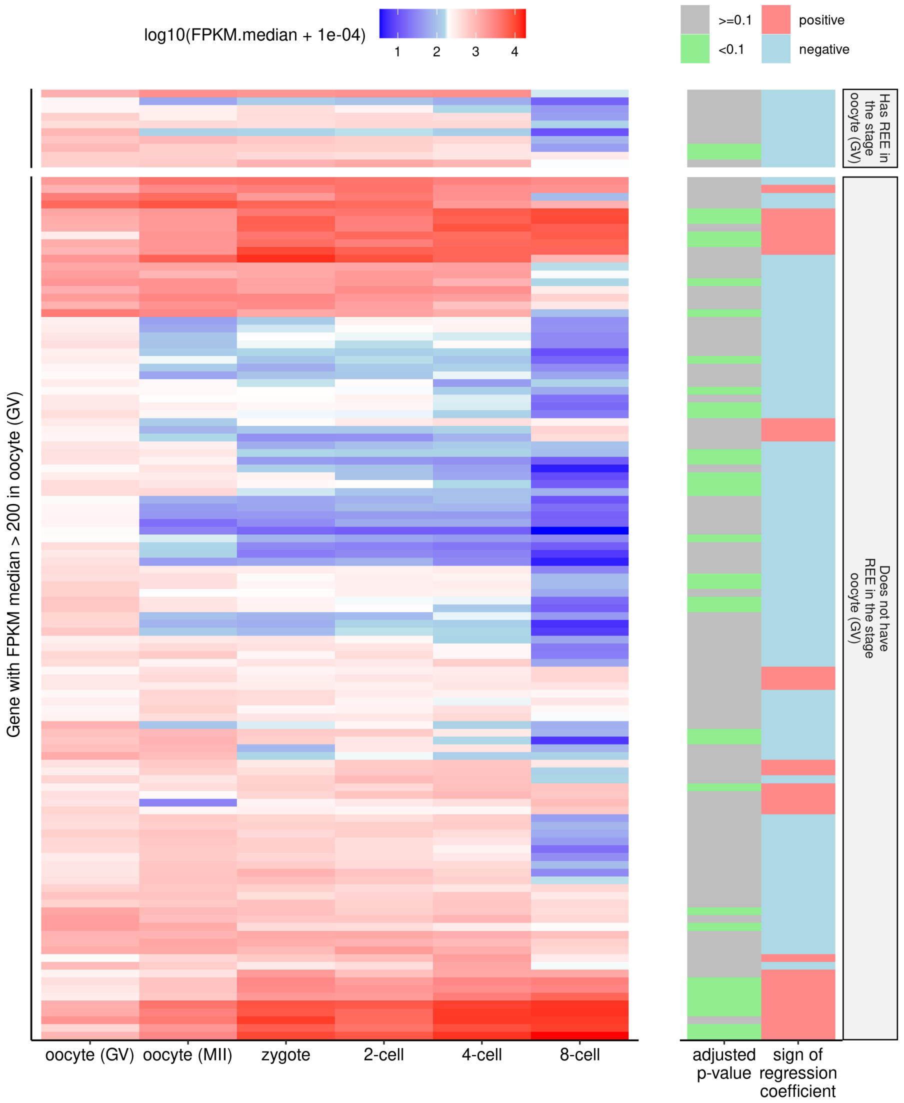

Supplementary Figure 21. An example heatmap for the gene FPKM median along the development progress, considering only those genes whose FPKM median is larger than 200 in the oocyte (GV) stage. Shown from left to right are the columns for log10(FPKM median + 1e-04) in oocytes (GV), oocytes (MII), zygotes,

2-cells, 4-cells, and 8-cells, and the binned Benjamini-Hochberg-adjusted adjusted *p*-values and signs of regression coefficients of the corresponding linear regression result for each gene. The regression of per-stage FPKM medians against stages was carried out by treating the stages (from oocyte (GV) to 8-cell) as the integers 1 to 6, respectively (and thus the "sample size" for each linear regression test is 6). The *p*-values are from the linear regression test with the null hypothesis being that the coefficient of stage for predicting the FPKM values is zero. We note that the prediction results are not very accurate due to the limited "sample size" (6) for each of the linear regression test; we ran the linear regression test mostly for displaying how well each gene follows a monotonically increasing (or decreasing) trend of expression change along the progressing of embryonic stages.

Supplementary Figure 22

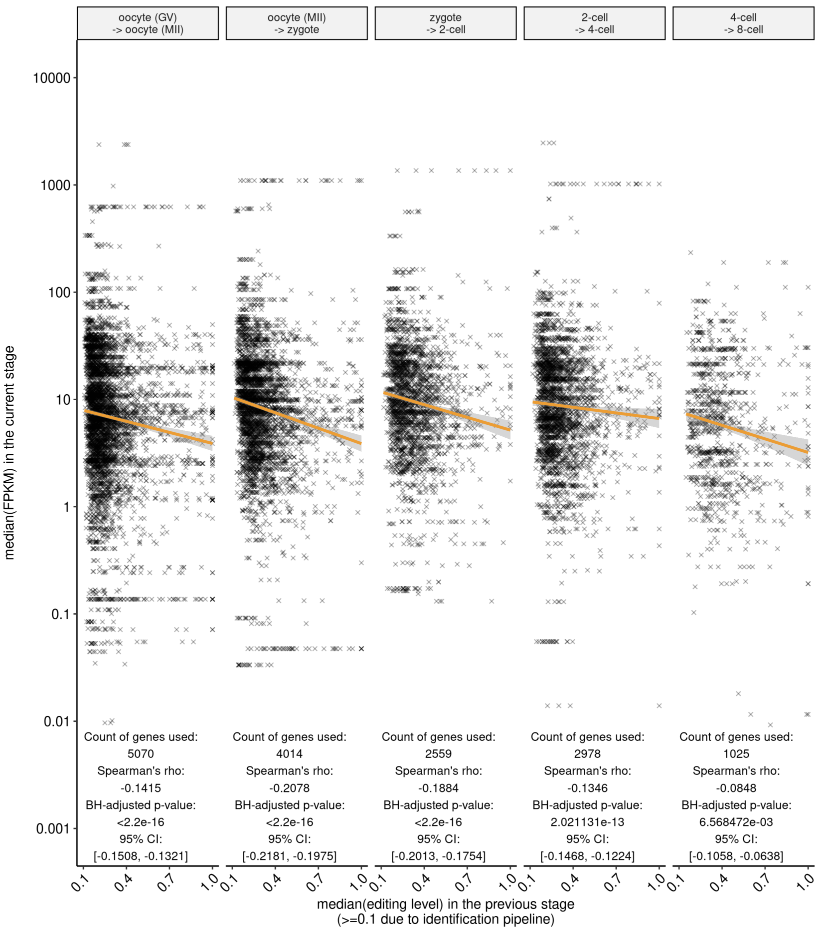

Supplementary Figure 22. Spearman's correlation coefficient between median editing level of a REE in a given stage and median FPKM of the gene hosting that REE in the latter stage. Only normal samples from early stages were considered. For each stage pair, only genes with median FPKM > 0.001 in the current stage were plotted. The *p*-value shown in each scatterplot is the Benjamini-Hochberg-adjusted (BH-adjusted) *p*-value for two-sided correlation test (computed by R's cor.test(method = "spearman")), with the null hypothesis being that the correlation is zero. The 95% confidence interval (CI) for this test was computed using the ci.spear function of the R

package statpsych. The trend of association is visualized by linear regression (with orange line denoting the fitted line and grey shadow denoting its 95% confidence interval of the slope of the regression, where the null hypothesis for the linear regression test is that the slope is zero).

Supplementary Figure 23

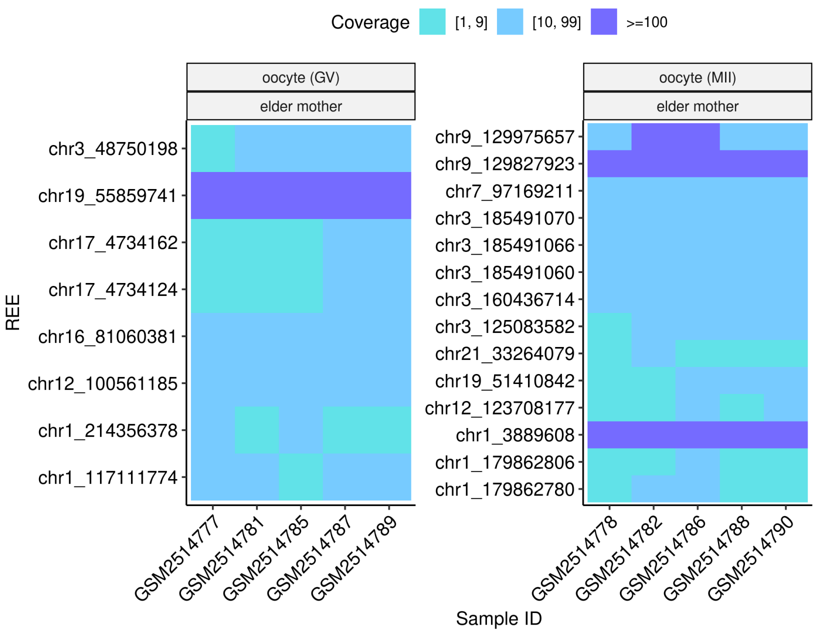

Supplementary Figure 23. Coverage of each of the 107 REE in the samples from samples of elder mothers. For each of oocyte types listed here, only those REEs determined to be completely lost were displayed.

Supplementary Figure 24

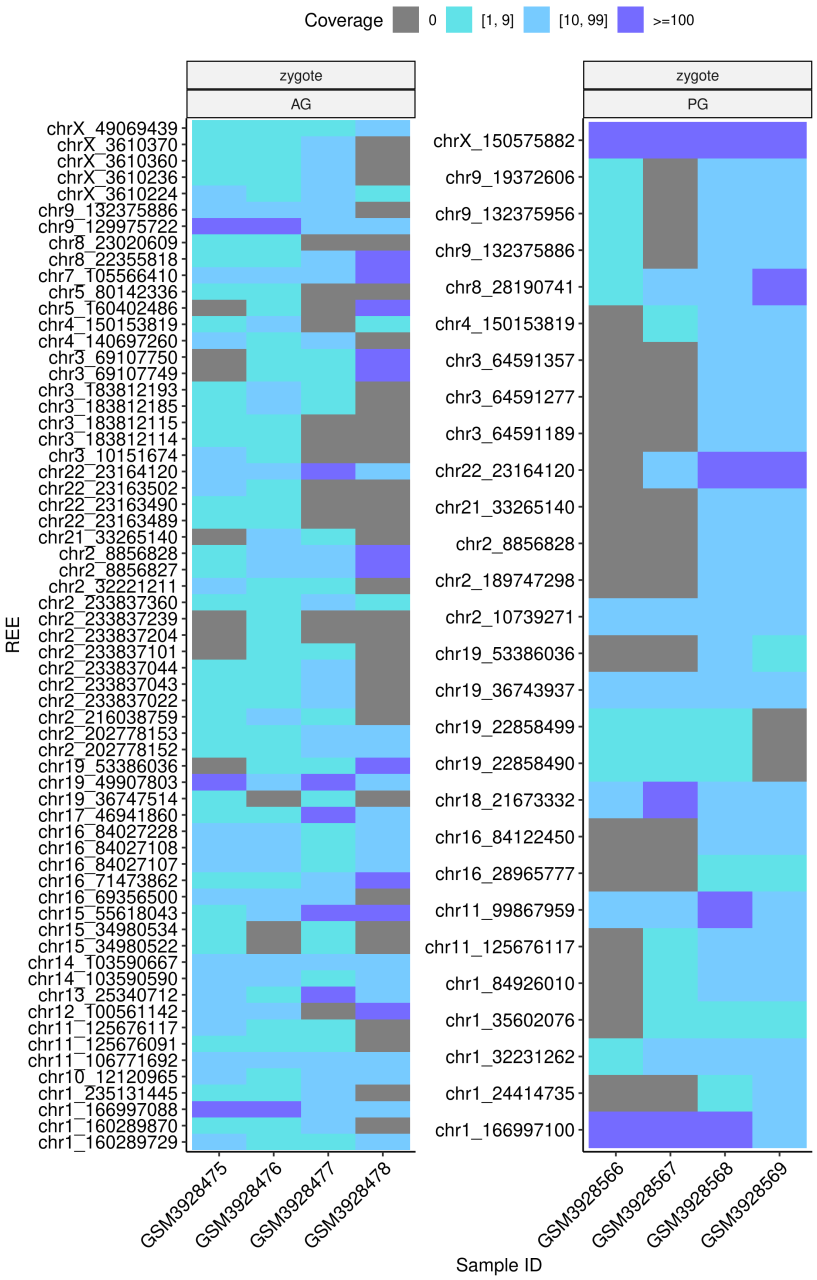

Supplementary Figure 24. Coverage of each of the 107 REE in the samples

from androgenetic/parthenogenetic zygotes. For each of embryo types listed here, only those REEs determined to be completely lost were displayed.

Supplementary Figure 25

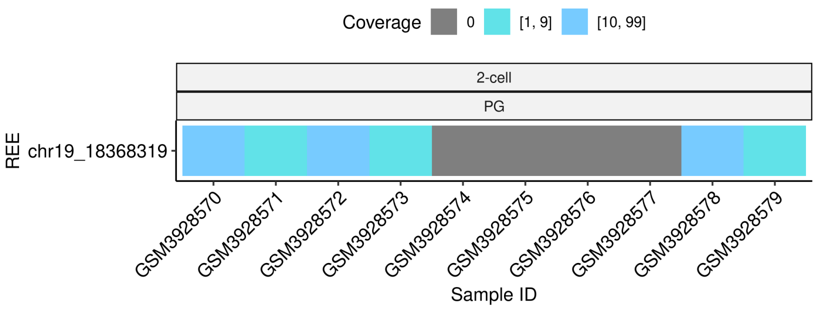

Supplementary Figure 25. Coverage of each of the 107 REE in the samples from parthenogenetic 2-cells. For each of embryo types listed here, only those REEs determined to be completely lost were displayed.

Supplementary Figure 26

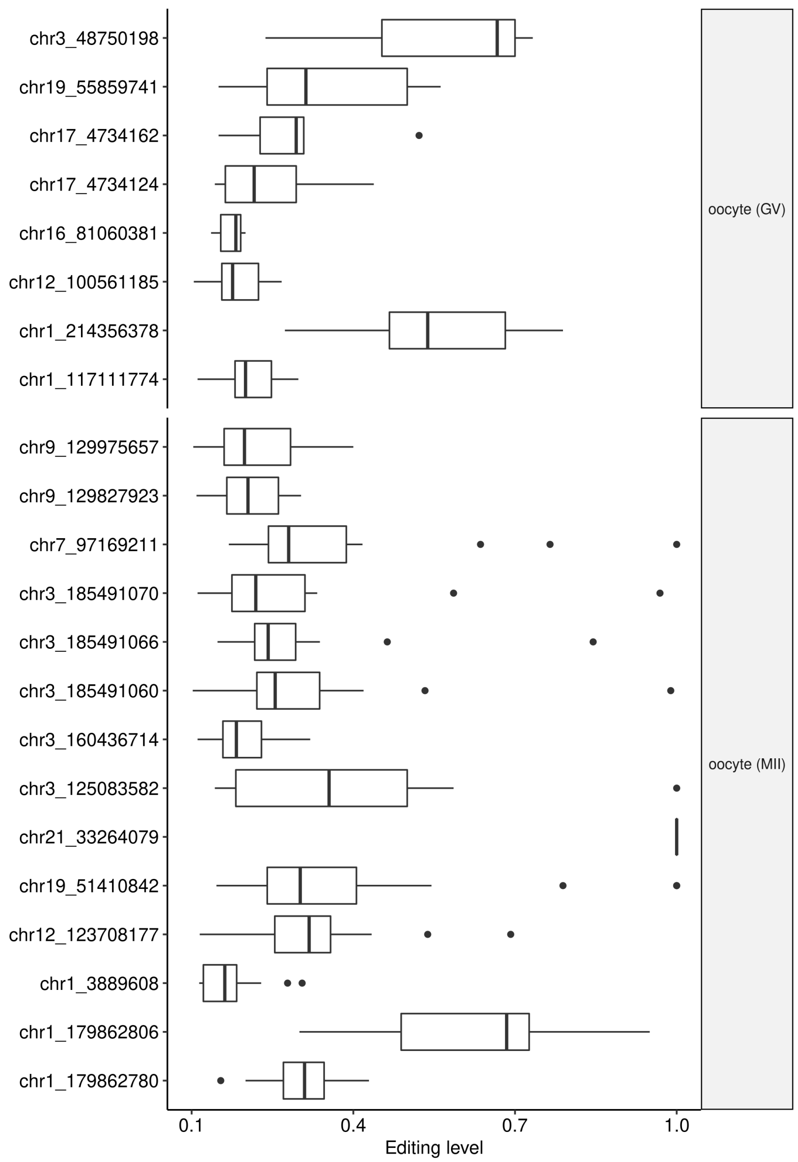

Supplementary Figure 26. Distribution of normal sample editing level of the 107 REEs lost in oocytes from elder mothers. We note that the editing site for chr21:33264079 is always 100%, and this site should be further examined with caution (also see Supplementary Figure 5 and Supplementary Figure 6). Symbols in boxplots

follow the definition by "geom_boxplot" of the R package "ggplot2"[33] : the inner thick line indicates the median; the lower and upper boundaries (or hinges) of the box indicate the first and third quartiles (i.e., 25% and 75% quantiles), respectively; the upper whisker extends from the hinge to the largest value no further than 1.5 * inter-quartile range (the third quartile minus the first quartile), the lower whisker extends from the hinge to the smallest value at most 1.5 * inter-quartile range of the hinge, and data beyond the end of the whiskers are the outlier points, which are plotted individually.

Supplementary Figure 27

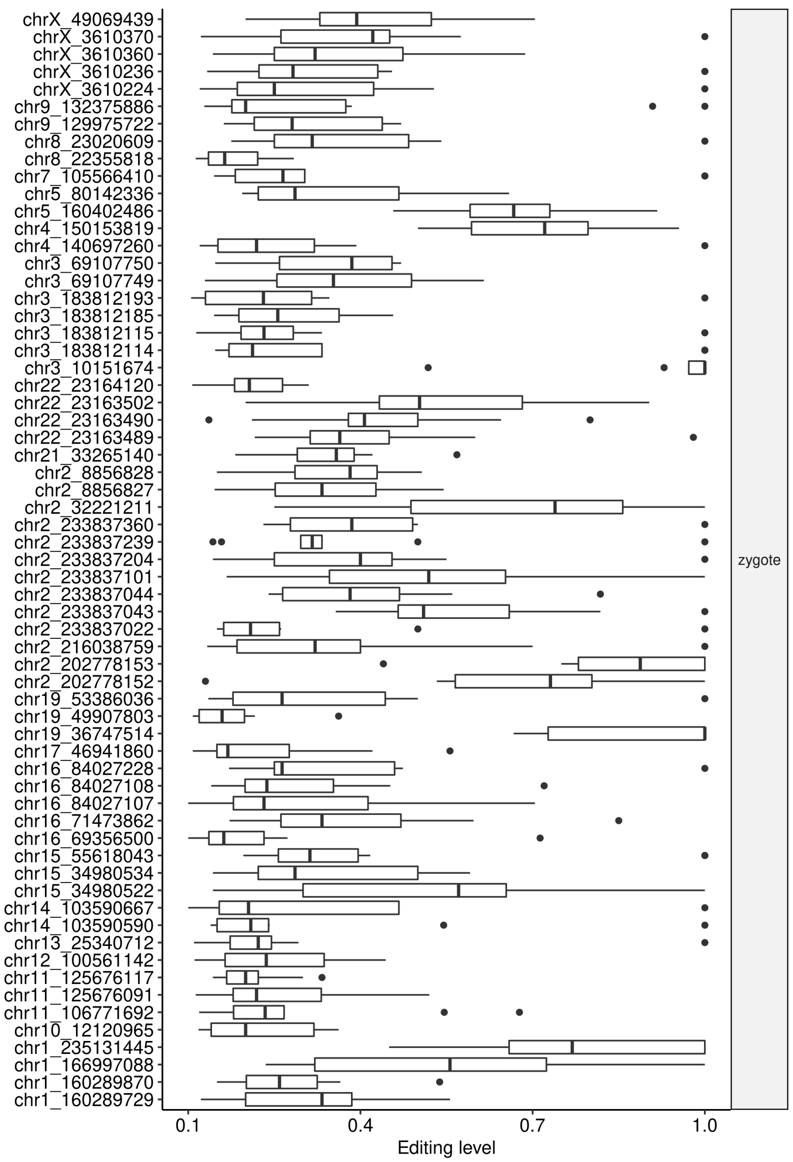

Supplementary Figure 27. Distribution of normal sample editing level of the 107 REEs lost in oocytes from androgenetic embryos. Symbols in boxplots follow the definition by "geom_boxplot" of the R package "ggplot2"[33] : the inner thick line indicates the median; the lower and upper boundaries (or hinges) of the box indicate the

first and third quartiles (i.e., 25% and 75% quantiles), respectively; the upper whisker extends from the hinge to the largest value no further than 1.5 * inter-quartile range (the third quartile minus the first quartile), the lower whisker extends from the hinge to the smallest value at most 1.5 * inter-quartile range of the hinge, and data beyond the end of the whiskers are the outlier points, which are plotted individually.

Supplementary Figure 28

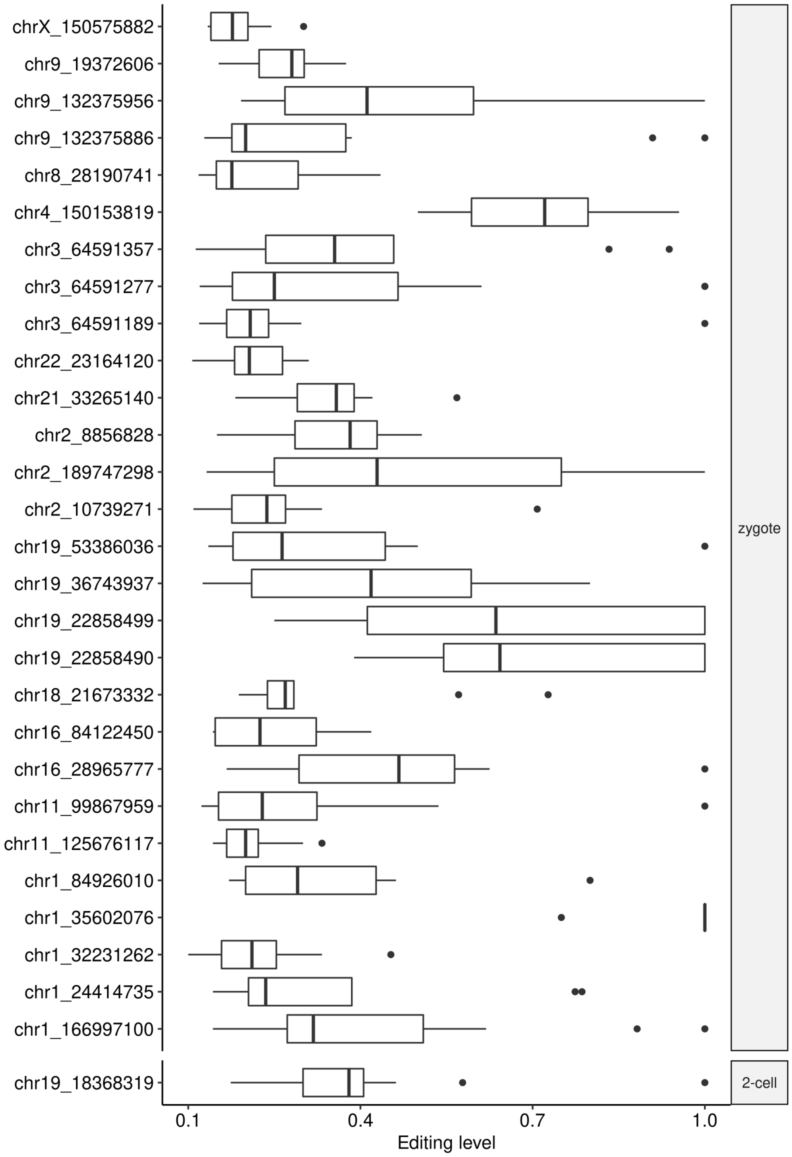

Supplementary Figure 28. Distribution of normal sample editing level of the 107 REEs lost in oocytes from parthenogenetic embryos. Symbols in boxplots follow the definition by "geom_boxplot" of the R package "ggplot2"[33] : the inner thick line indicates the median; the lower and upper boundaries (or hinges) of the box indi-

cate the first and third quartiles (i.e., 25% and 75% quantiles), respectively; the upper whisker extends from the hinge to the largest value no further than 1.5 * inter-quartile range (the third quartile minus the first quartile), the lower whisker extends from the hinge to the smallest value at most 1.5 * inter-quartile range of the hinge, and data beyond the end of the whiskers are the outlier points, which are plotted individually.

Supplementary Figure 29

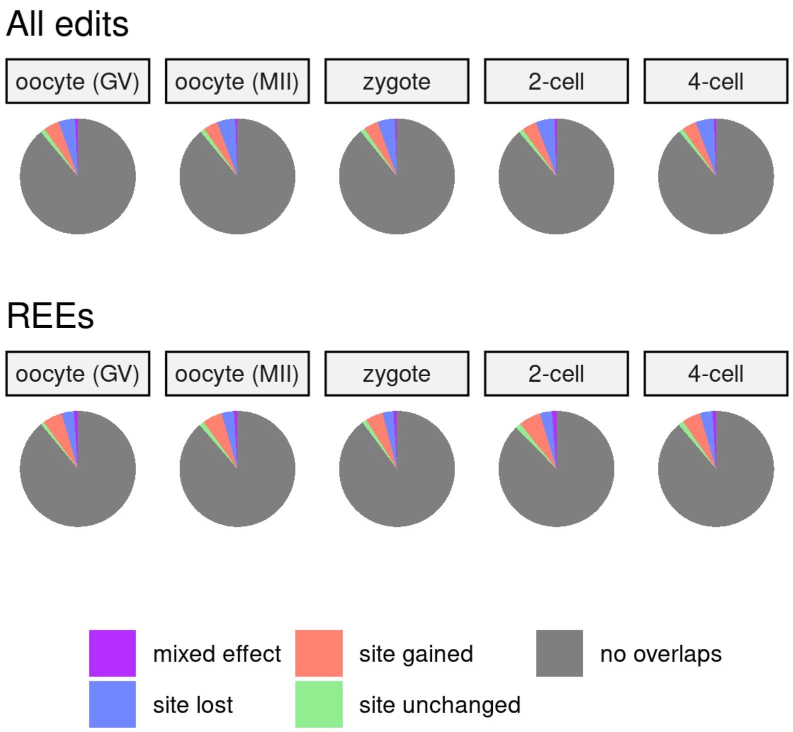

Supplementary Figure 29. Ratio of MBS-altering edits in 3'-UTR REEs and all 3'-UTR edits, with no-overlap edits considered.

Supplementary Figure 30

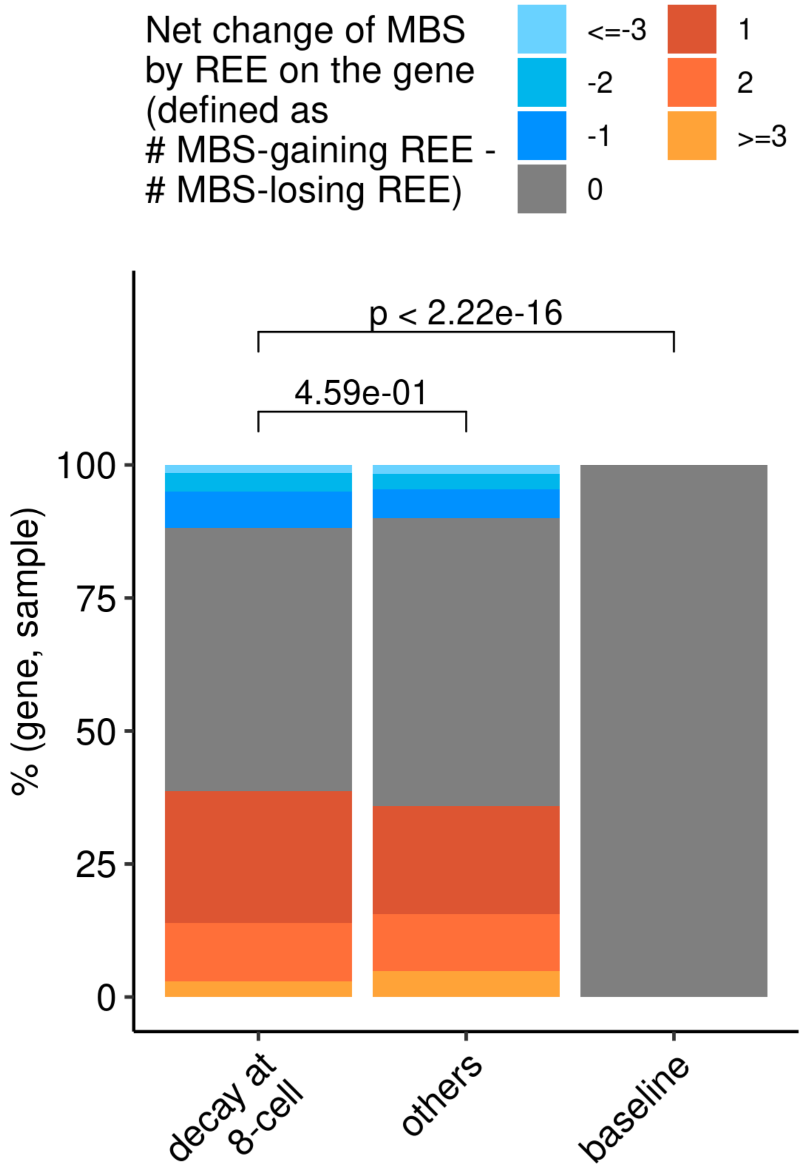

Supplementary Figure 30. Genes targeted by maternal mRNA clearance ex-

hibited a more positive net change in MBS counts due to REEs than did baseline but not did other maternal genes. All *p*-values were unadjusted and were derived from one-tailed, unpaired Wilcoxon's Rank Sum test, with the alternative hypothesis being that the median value for the left ("decay at 8-cell"; see the subsection "Annotation of maternal genes and targets of maternal mRNA clearance" in Methods for more details about "decay at 8-cell" and "others") group would be greater than that for the right (others/baseline) group. The baseline group is just a value of 0; we plotted it here for the sake of visual clarity. The unpaired test between "decay at 8-cell" and "others" involves 17,060 and 17,755 pairs of (gene, sample) for "decay at 8-cell" and "others", respectively, and its estimated (pseudo)median and 95 percent confidence interval reported by R are 7.537961×10^{-6} and $[-3.372651 \times 10^{-6}, +\infty)$, respectively. The test between "decay at 8-cell" and the 0 baseline involves 17,060 pairs of (gene, sample) for "decay at 8-cell", and has an estimated (pseudo)median of 0.9999568 and a 95 percent confidence interval of $[0.9999797, +\infty)$ as reported by R.

Supplementary Figure 31

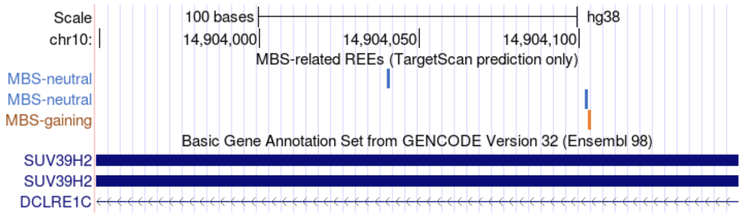

Supplementary Figure 31. An example of REE-induced MBS gains on a known clearance target *SUV39H2*. We note that this MBS-gaining prediction is not supported by miRanda. The genome assembly is hg38 and the gene annotation is GENCODE version 32.

Supplementary Figure 32

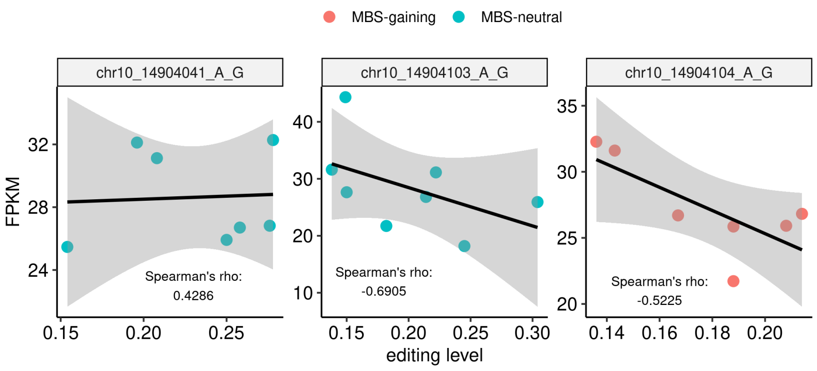

Supplementary Figure 32. Scatterplot for the editing level of MBS-related REE in oocyte (GV) on *SUV39H2* in each sample and the FPKM of *SUV39H2* in the same sample. Note that we only examined for each REE those oocyte (GV) samples where this REE was detected; therefore, points for different REEs correspond to different sample subsets, and their FPKM might not match completely. The trend of association is visualized by linear regression (with line denoting the fitted line and grey shadow denoting the 95% confidence interval). We also note that the chr10_14904104_A_G REE was predicted to be MBS-gaining by TargetScan, but not by miRanda.

Supplementary Figure 33

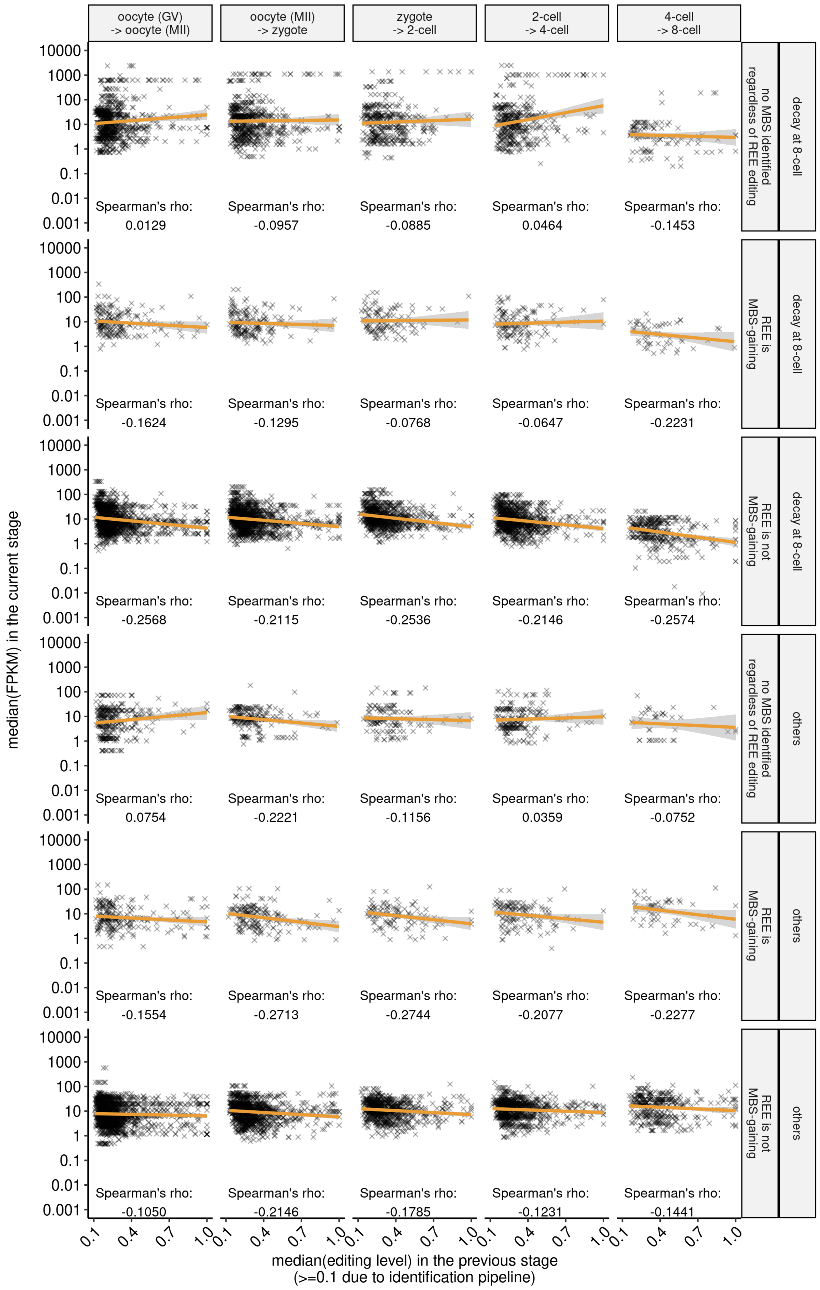

Supplementary Figure 33. Spearman's correlation coefficient between median

editing level of a REE in a given stage and median FPKM of the gene hosting that REE in the latter stage, with (gene, REE) pairs grouped by how the REE is related to MBS. Only normal samples from early stages were considered. For each stage pair, only genes with FPKM > 0.001 were plotted. The trend of association is visualized by linear regression (with orange line denoting the fitted line and grey shadow denoting the 95% confidence interval).

Supplementary Figure 34

a

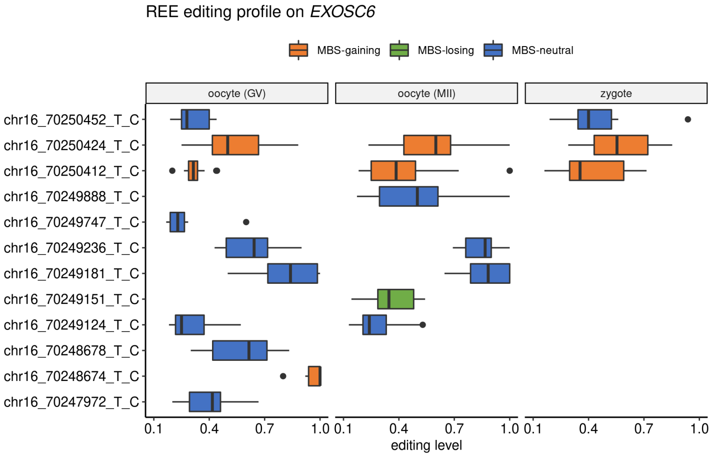

b

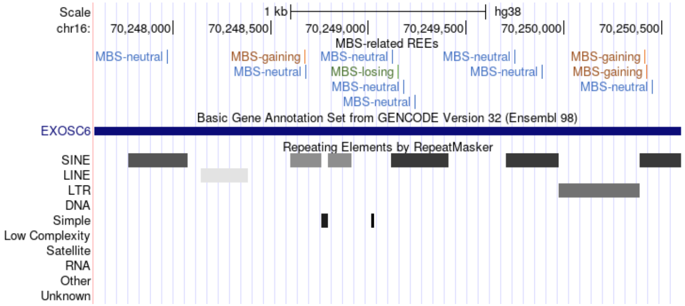

c

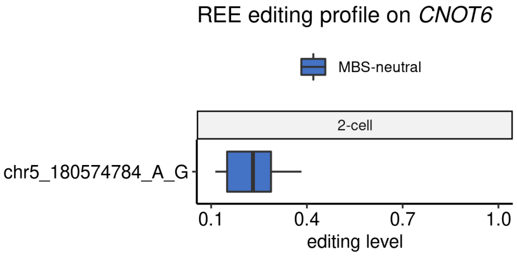

Supplementary Figure 34. *EXOSC6* and *CNOT6* are the REE-targeted genes of the RNA degradation pathways in question. (a), editing profile of REEs on

EXOSC6 across oocyte (GV), oocyte (MII), and zygote stages. (b), UCSC Genome Browser track of *EXOSC6* -targeting REEs. The genome assembly is hg38 and the gene annotation is GENCODE version 32. (c), editing profile of the single REE on *CNOT6* in the 2-cell stage. Symbols in boxplots follow the definition by "geom_boxplot" of the R package "ggplot2"[33] : the inner thick line indicates the median; the lower and upper boundaries (or hinges) of the box indicate the first and third quartiles (i.e., 25% and 75% quantiles), respectively; the upper whisker extends from the hinge to the largest value no further than 1.5 * inter-quartile range (the third quartile minus the first quartile), the lower whisker extends from the hinge to the smallest value at most 1.5 * inter-quartile range of the hinge, and data beyond the end of the whiskers are the outlier points, which are plotted individually.

Supplementary Figure 35

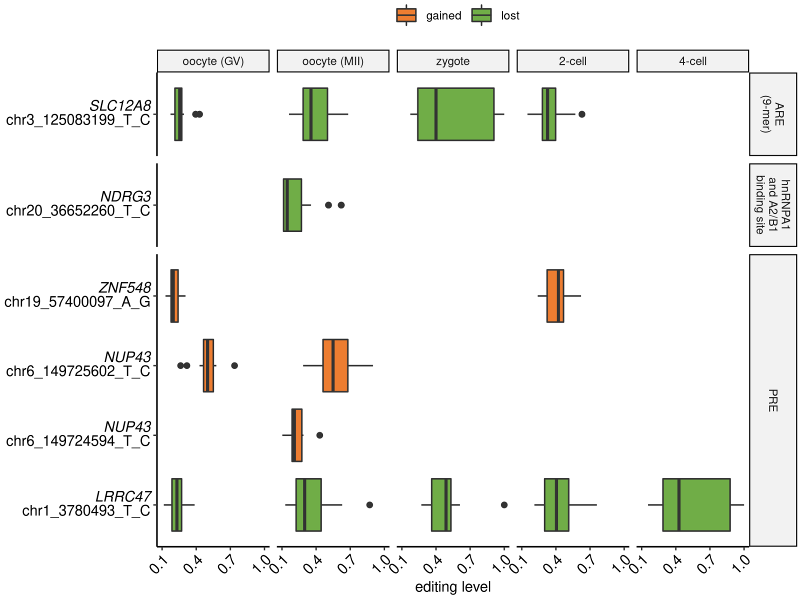

Supplementary Figure 35. Editing profile for normal early stage REEs that alter the CCR4-NOT sequence motifs. Symbols in boxplots follow the definition by "geom_boxplot" of the R package "ggplot2"[33] : the inner thick line indicates the median; the lower and upper boundaries (or hinges) of the box indicate the first and third quartiles (i.e., 25% and 75% quantiles), respectively; the upper whisker extends from the hinge to the largest value no further than 1.5 * inter-quartile range (the third quartile minus the first quartile), the lower whisker extends from the hinge to the smallest value at most 1.5 * inter-quartile range of the hinge, and data beyond the end of the whiskers are the outlier points, which are plotted individually.

Supplementary Figure 36

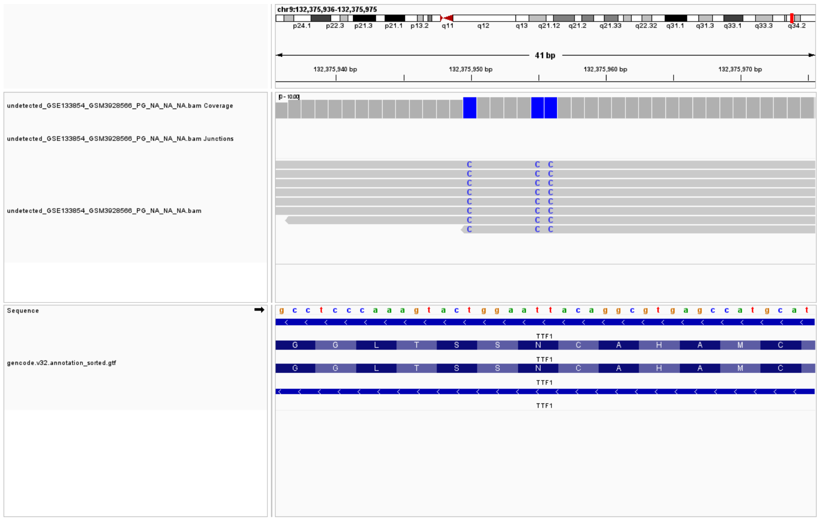

Supplementary Figure 36. IGV tracks showing read alignment at the *TTF1* recoding edit (chr9:132,375,956) in the PG zygote GSM3928566 from the dataset GSE133854. The genome assembly is hg38 and the gene annotation is GENCODE version 32.

Supplementary Figure 37

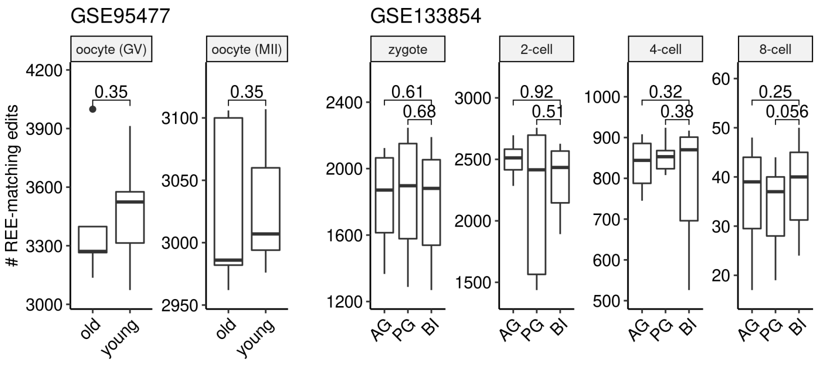

Supplementary Figure 37. Distribution of REE-matching edits on protein-coding genes in datasets covering abnormal embryos and embryos from elder mothers. All *p*-values were unadjusted and were derived from one-tailed, unpaired Wilcoxon's Rank Sum tests, with the alternative hypothesis being that the median value for the left (old mother/AG/PG) group would be less than that for the right (young mother/control/BI (biparental embryos)) group. See Supplementary Data 29 for description of sample size, difference in location (the effect size for Wilcoxon's Rank Sum test), and its 95% confidence interval for each of these tests. Symbols in boxplots follow the definition by "geom_boxplot" of the R package "ggplot2"[33] : the inner thick line indicates the median; the lower and upper boundaries (or hinges) of the box indicate the first and third quartiles (i.e., 25% and 75% quantiles), respectively; the upper whisker extends from the hinge to the largest value no further than 1.5 * inter-quartile range (the third quartile minus the first quartile), the lower whisker extends from the hinge to the smallest value at most 1.5 * inter-quartile range of the hinge, and data beyond the end of the whiskers are the outlier points, which are plotted individually.

Supplementary Figure 38

a

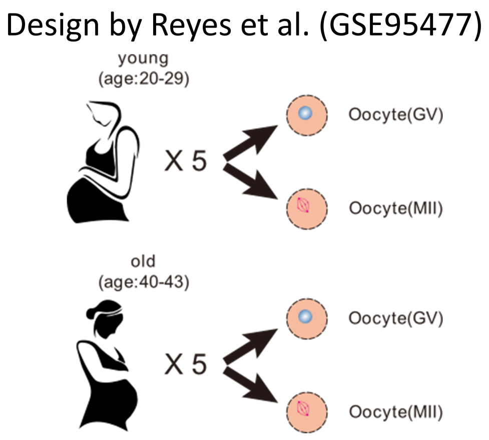

b

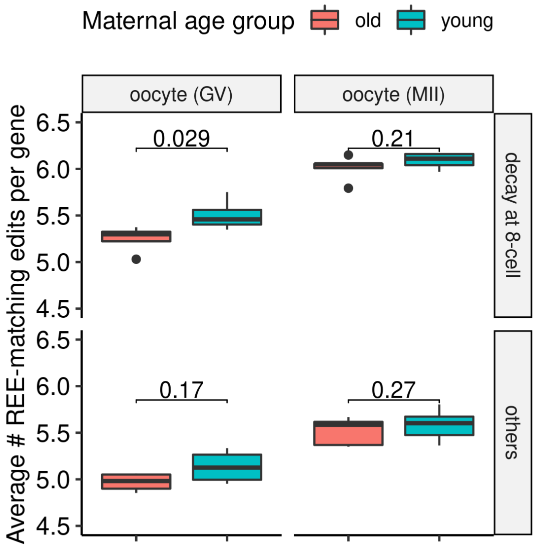

Supplementary Figure 38. Number of REE-matching edits per gene in different stages of GSE95477 samples and different gene groups. (a) Design for the

study of dataset GSE95477[32]. (b) Average numbers of REE-matching edits per gene in different stages and gene groups. All *p*-values were unadjusted and were derived from one-tailed, unpaired Wilcoxon's Rank Sum tests, with the alternative hypothesis being that the median of "average number of REE-matching edits per gene" would decrease from young to old mothers. See the subsection "Annotation of maternal genes and targets of maternal mRNA clearance" in Methods for more details about "decay at 8-cell" and "others". Note that some outlier samples are excluded from this plot (Supplementary Note 7, Supplementary Figure 39, and Supplementary Figure 40; see also Supplementary Figure 41 for the analysis of other datasets). See Supplementary Data 30 for description of sample size, difference in location (the effect size for Wilcoxon's Rank Sum test), and its 95% confidence interval for each of these tests. Symbols in boxplots follow the definition by "geom_boxplot" of the R package "ggplot2"[33] : the inner thick line indicates the median; the lower and upper boundaries (or hinges) of the box indicate the first and third quartiles (i.e., 25% and 75% quantiles), respectively; the upper whisker extends from the hinge to the largest value no further than 1.5 * inter-quartile range (the third quartile minus the first quartile), the lower whisker extends from the hinge to the smallest value at most 1.5 * inter-quartile range of the hinge, and data beyond the end of the whiskers are the outlier points, which are plotted individually.

Supplementary Figure 39

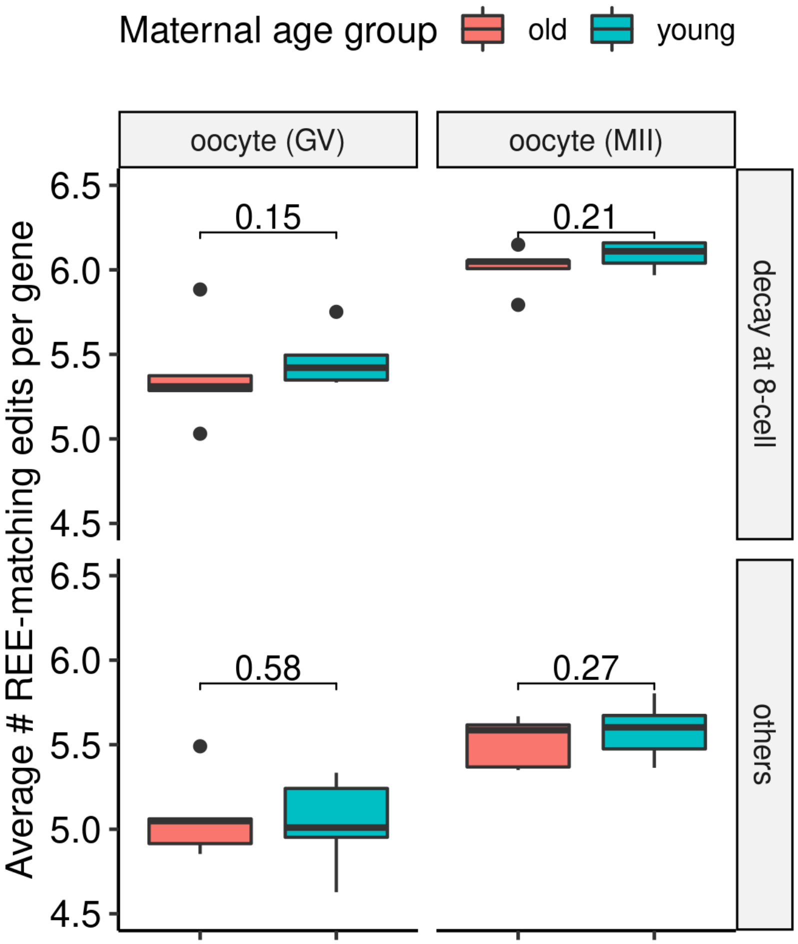

Supplementary Figure 39. Results from the same analysis whose results are illustrated in Supplementary Figure 38b, but with the inclusion of all samples from this dataset. All *p*-values were unadjusted and were derived from one-tailed, unpaired Wilcoxon's Rank Sum tests, with the alternative hypothesis being that the average number of REE-matching edits per gene would decrease from young to old mothers. See Supplementary Data 31 for description of sample size, difference in

location (the effect size for Wilcoxon's Rank Sum test), and its 95% confidence interval for each of these tests. Symbols in boxplots follow the definition by "geom_boxplot" of the R package "ggplot2"[33] : the inner thick line indicates the median; the lower and upper boundaries (or hinges) of the box indicate the first and third quartiles (i.e., 25% and 75% quantiles), respectively; the upper whisker extends from the hinge to the largest value no further than 1.5 * inter-quartile range (the third quartile minus the first quartile), the lower whisker extends from the hinge to the smallest value at most 1.5 * inter-quartile range of the hinge, and data beyond the end of the whiskers are the outlier points, which are plotted individually.

Supplementary Figure 40

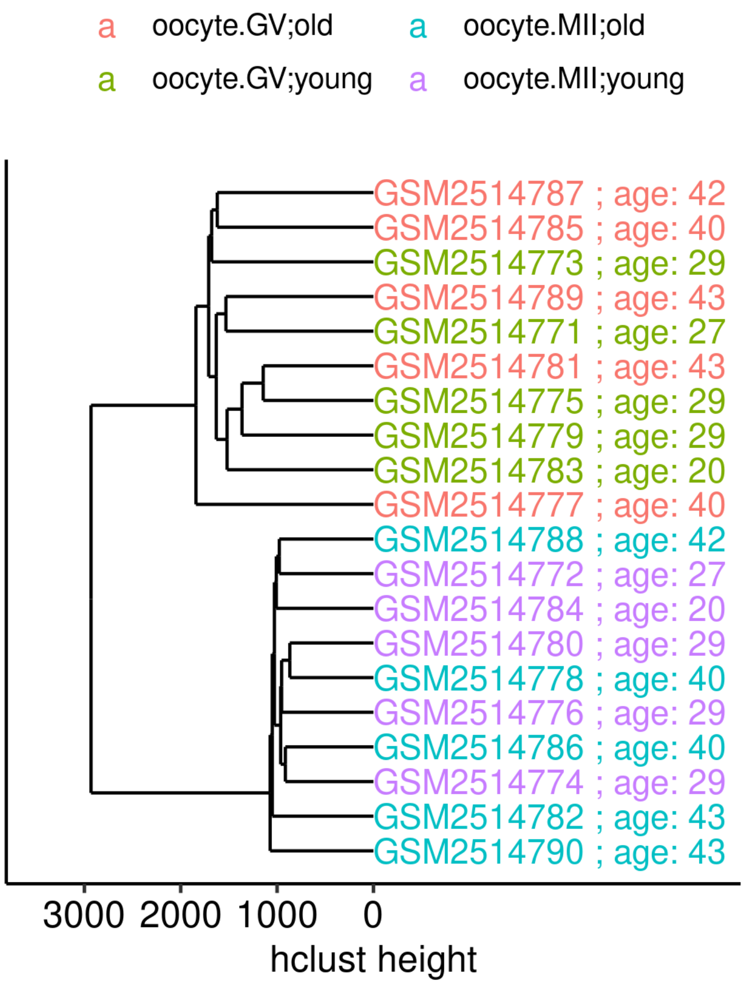

Supplementary Figure 40. Hierarchical clustering of all samples from GSE95477 using the R function "hclust", with the Manhattan distance based on all REE-matching edits of maternal genes.

Supplementary Figure 41

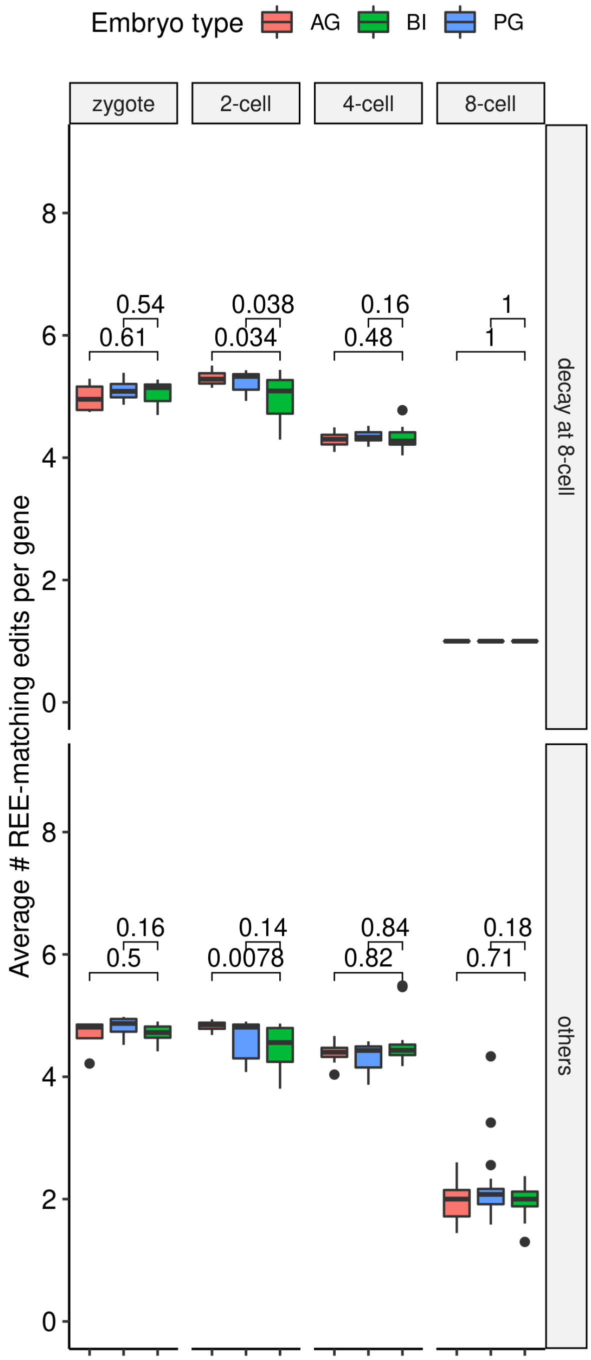

Supplementary Figure 41. Results from the same analysis whose results are illustrated in Supplementary Figure 37, but conducted with other datasets containing data on abnormal early embryos (the GSE133854 dataset). All *p*-values were unadjusted and based on one-tailed, unpaired Wilcoxon's Rank Sum test, with the alternative hypothesis being that the left (abnormal) group is greater than the right (control) group. AG, androgenetic; PG, parthenogenetic; BI, biparental (i.e., normal). See Supplementary Data 32 for description of sample size, difference in location (the effect size for Wilcoxon's Rank Sum test), and its 95% confidence interval for each of these tests (except for the tests in the "decay at 8-cell" vs. "8-cell" group, where all points are of the same value (possibly partly due to the depletion of such genes in the 8-cell stage) and the difference in location and the confidence interval cannot be estimated). Symbols in boxplots follow the definition by "geom_boxplot" of the R package "ggplot2"[33] : the inner thick line indicates the median; the lower and upper boundaries (or hinges) of the box indicate the first and third quartiles (i.e., 25% and 75% quantiles), respectively; the upper whisker extends from the hinge to the largest value no further than 1.5 * inter-quartile range (the third quartile minus the first quartile), the lower whisker extends from the hinge to the smallest value at most 1.5 * inter-quartile range of the hinge, and data beyond the end of the whiskers are the outlier points, which are plotted individually.

Supplementary Figure 42

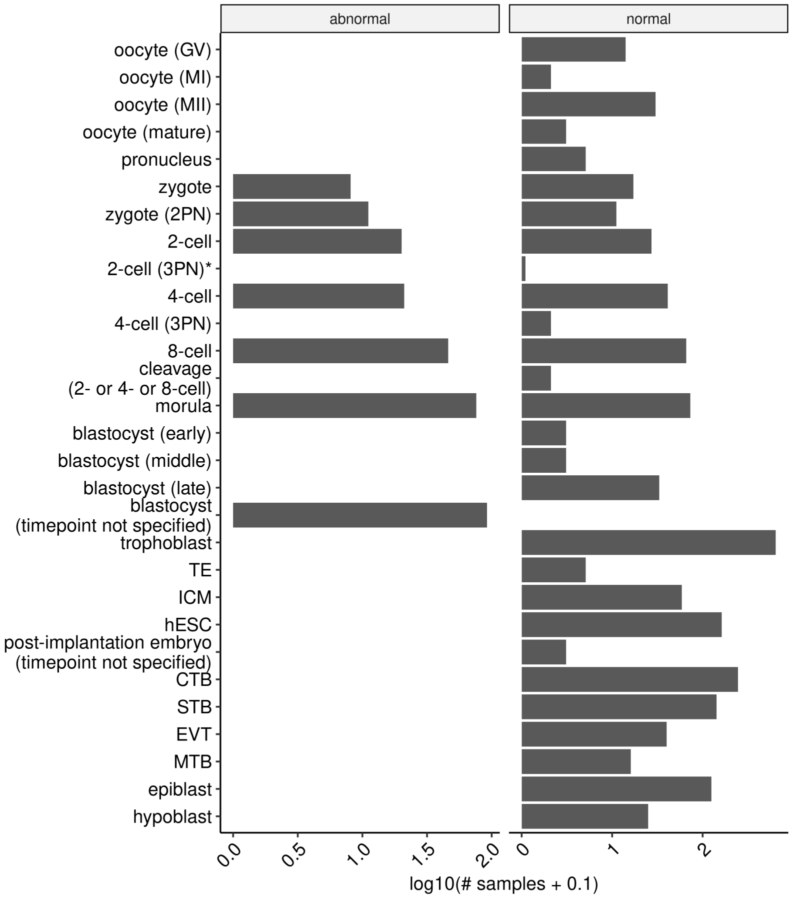

Supplementary Figure 42. Count of normal or abnormal samples (in log10 scale) in each stage. Groups labeled with * have only one sample. GV, germinal vesicle. MI, metaphase of first meiosis. MII, metaphase of second meiosis. PN, pronuclear. TE, trophectoderm. ICM, inner cell mass. hESC, human embryonic stem cell. CTB, cytotrophoblast. STB, syncytiotrophoblast. EVT, extravillous trophoblast. MTB, migratory trophoblast.

Supplementary Figure 43

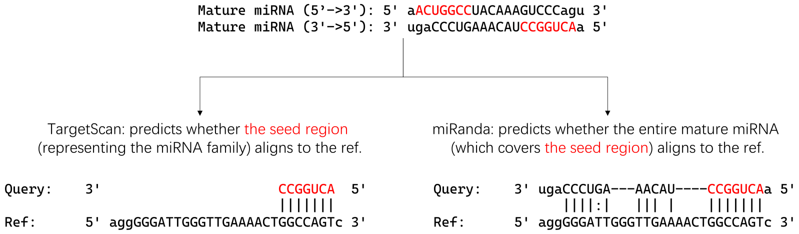

Supplementary Figure 43. How TargetScan and miRanda make their predictions.

Supplementary Figure 44

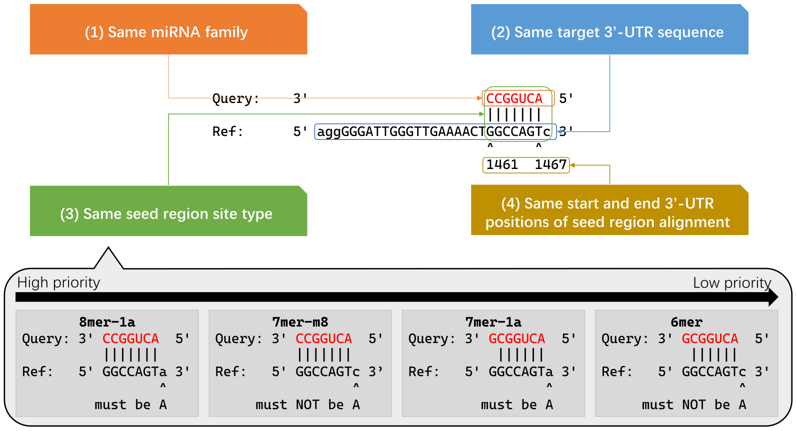

Supplementary Figure 44. Criteria for collapsing miRanda predictions and also for determining the intersection of TargetScan and (collapsed) miRanda predictions.

Supplementary References

[1] G. Ramaswami, R. Zhang, R. Piskol, L. P. Keegan, P. Deng, M. A. O'Connell, and J. B. Li. Identifying RNA editing sites using RNA sequencing data alone. *Nat Methods*, 10:128–32, 2013. doi: 10.1038/nmeth.2330.

[2] A. Frankish, M. Diekhans, A. M. Ferreira, R. Johnson, I. Jungreis, J. Loveland, J. M. Mudge, C. Sisu, J. Wright, J. Armstrong, I. Barnes, A. Berry, A. Bignell, S. Carbonell Sala, J. Chrast, F. Cunningham, T. Di Domenico, S. Donaldson, I. T. Fiddes, C. García Girón, J. M. Gonzalez, T. Grego, M. Hardy, T. Hourlier, T. Hunt, O. G. Izuogu, J. Lagarde, F. J. Martin, L. Martínez, S. Mohanan, P. Muir, F. C. P. Navarro, A. Parker, B. Pei, F. Pozo, M. Ruffier, B. M. Schmitt, E. Stapleton, M. M. Suner, I. Sycheva, B. Uszczynska-Ratajczak, J. Xu, A. Yates, D. Zerbino, Y. Zhang, B. Aken, J. S. Choudhary, M. Gerstein, R. Guigó, T. J. P. Hubbard, M. Kellis, B. Paten, A. Reymond, M. L. Tress, and P. Flicek. GENCODE reference annotation for the human and mouse genomes. *Nucleic Acids Res*, 47:D766–D773, 2019. doi: 10.1093/nar/gky955.

[3] Marcel Martin. Cutadapt removes adapter sequences from high-throughput sequencing reads. *EMBnet.journal*, 17(1):10–12, 2011. ISSN 2226-6089. doi: 10.14806/ej.17.1.200. URL `http://journal.embnet.org/index.php/embnetjournal/article/view/200`.

[4] S. Chen, Y. Zhou, Y. Chen, and J. Gu. fastp: an ultra-fast all-in-one FASTQ preprocessor. *Bioinformatics*, 34:i884–i890, 2018. doi: 10.1093/bioinformatics/bty560.

[5] H. Li and R. Durbin. Fast and accurate short read alignment with Burrows-Wheeler transform. *Bioinformatics*, 25:1754–60, 2009. doi: 10.1093/bioinformatics/btp324.

[6] M. A. DePristo, E. Banks, R. Poplin, K. V. Garimella, J. R. Maguire, C. Hartl, A. A. Philippakis, G. del Angel, M. A. Rivas, M. Hanna, A. McKenna, T. J. Fennell, A. M. Kernytsky, A. Y. Sivachenko, K. Cibulskis, S. B. Gabriel, D. Altshuler, and M. J. Daly. A framework for variation discovery and genotyping using next-generation DNA sequencing data. *Nat Genet*, 43:491–8, 2011. doi: 10.1038/ng.806.

[7] S. T. Sherry, M. H. Ward, M. Kholodov, J. Baker, L. Phan, E. M. Smigielski, and K. Sirotkin. dbSNP: the NCBI database of genetic variation. *Nucleic Acids Res*, 29:308–11, 2001. doi: 10.1093/nar/29.1.308.

[8] C. Lo Giudice, M. A. Tangaro, G. Pesole, and E. Picardi. Investigating RNA editing in deep transcriptome datasets with REDItools and REDIportal. *Nat Protoc*, 15:1098–1131, 2020. doi: 10.1038/s41596-019-0279-7.

[9] 1000 Genomes Project Consortium, A. Auton, L. D. Brooks, R. M. Durbin, E. P. Garrison, H. M. Kang, J. O. Korbel, J. L. Marchini, S. McCarthy, G. A. McVean, and G. R. Abecasis. A global reference for human genetic variation. *Nature*, 526: 68–74, 2015. doi: 10.1038/nature15393.

[10] K. J. Karczewski, L. C. Francioli, G. Tiao, B. B. Cummings, J. Alföldi, Q. Wang, R. L. Collins, K. M. Laricchia, A. Ganna, D. P. Birnbaum, L. D. Gauthier, H. Brand, M. Solomonson, N. A. Watts, D. Rhodes, M. Singer-Berk, E. M. England, E. G. Seaby, J. A. Kosmicki, R. K. Walters, K. Tashman, Y. Farjoun, E. Banks, T. Poterba, A. Wang, C. Seed, N. Whiffin, J. X. Chong, K. E. Samocha, E. Pierce-Hoffman, Z. Zappala, A. H. O'Donnell-Luria, E. V. Minikel, B. Weisburd, M. Lek, J. S. Ware, C. Vittal, I. M. Armean, L. Bergelson, K. Cibulskis, K. M. Connolly, M. Covarrubias, S. Donnelly, S. Ferriera, S. Gabriel, J. Gentry, N. Gupta, T. Jeandet, D. Kaplan, C. Llanwarne, R. Munshi, S. Novod, N. Petrillo, D. Roazen, V. Ruano-Rubio, A. Saltzman, M. Schleicher, J. Soto, K. Tibbetts,

C. Tolonen, G. Wade, M. E. Talkowski, Genome Aggregation Database Consortium, B. M. Neale, M. J. Daly, and D. G. MacArthur. The mutational constraint spectrum quantified from variation in 141,456 humans. *Nature*, 581:434–443, 2020. doi: 10.1038/s41586-020-2308-7.

[11] L Phan, Y Jin, H Zhang, W Qiang, D Shekhtman, D Shao, D Revoe, R Villamarin, E Ivanchenko, M Kimura, Z. Y Wang, L Hao, N Sharopova, M Bihan, A Sturcke, M Lee, N Popova, W Wu, C Bastiani, M Ward, J. B Holmes, V Lyoshin, K Kaur, E Moyer, M Feolo, and B. L Kattman. ALFA: Allele Frequency Aggregator, March 2020. URL `https://www.ncbi.nlm.nih.gov/snp/docs/gsr/alfa/`.

[12] E. Y. Levanon, M. Hallegger, Y. Kinar, R. Shemesh, K. Djinovic-Carugo, G. Rechavi, M. F. Jantsch, and E. Eisenberg. Evolutionarily conserved human targets of adenosine to inosine RNA editing. *Nucleic Acids Res*, 33:1162–8, 2005. doi: 10.1093/nar/gki239.

[13] R. Shtrichman, I. Germanguz, R. Mandel, A. Ziskind, I. Nahor, M. Safran, S. Osenberg, O. Sherf, G. Rechavi, and J. Itskovitz-Eldor. Altered A-to-I RNA editing in human embryogenesis. *PLoS One*, 7:e41576, 2012. doi: 10.1371/journal.pone.0041576.

[14] X. Hu, S. Wan, Y. Ou, B. Zhou, J. Zhu, X. Yi, Y. Guan, W. Jia, X. Liu, Q. Wang, Y. Qi, Q. Yuan, W. Huang, W. Liao, Y. Wang, Q. Zhang, H. Xiao, X. Chen, and J. Huang. RNA over-editing of BLCAP contributes to hepatocarcinogenesis identified by whole-genome and transcriptome sequencing. *Cancer Lett*, 357: 510–9, 2015. doi: 10.1016/j.canlet.2014.12.006.

[15] P. V. Kharchenko, L. Silberstein, and D. T. Scadden. Bayesian approach to single-cell differential expression analysis. *Nat Methods*, 11:740–2, 2014. doi: 10.1038/nmeth.2967.

[16] J. Ding, C. Lin, and Z. Bar-Joseph. Cell lineage inference from SNP and scRNA-Seq data. *Nucleic Acids Res*, 47:e56, 2019. doi: 10.1093/nar/gkz146.

[17] V. Zachariadis, H. Cheng, N. Andrews, and M. Enge. A Highly Scalable Method for Joint Whole-Genome Sequencing and Gene-Expression Profiling of Single Cells. *Mol Cell*, 80:541–553.e5, 2020. doi: 10.1016/j.molcel.2020.09.025.

[18] I. C. Macaulay, W. Haerty, P. Kumar, Y. I. Li, T. X. Hu, M. J. Teng, M. Goolam, N. Saurat, P. Coupland, L. M. Shirley, M. Smith, N. Van der Aa, R. Banerjee, P. D. Ellis, M. A. Quail, H. P. Swerdlow, M. Zernicka-Goetz, F. J. Livesey, C. P. Ponting, and T. Voet. G&T-seq: parallel sequencing of single-cell genomes and transcriptomes. *Nat Methods*, 12:519–22, 2015. doi: 10.1038/nmeth.3370.

[19] S. H. Roth, E. Y. Levanon, and E. Eisenberg. Genome-wide quantification of ADAR adenosine-to-inosine RNA editing activity. *Nat Methods*, 16:1131–1138, 2019. doi: 10.1038/s41592-019-0610-9.

[20] Q. Q. Sha, W. Zheng, Y. W. Wu, S. Li, L. Guo, S. Zhang, G. Lin, X. H. Ou, and H. Y. Fan. Dynamics and clinical relevance of maternal mRNA clearance during the oocyte-to-embryo transition in humans. *Nat Commun*, 11:4917, 2020. doi: 10.1038/s41467-020-18680-6.

[21] J. Ma, Y. Fukuda, and R. M. Schultz. Mobilization of Dormant Cnot7 mRNA Promotes Deadenylation of Maternal Transcripts During Mouse Oocyte Maturation. *Biol Reprod*, 93:48, 2015. doi: 10.1095/biolreprod.115.130344.

[22] N. Chalabi Hagkarim and R. J. Grand. The Regulatory Properties of the Ccr4-Not Complex. *Cells*, 9, 2020. doi: 10.3390/cells9112379.

[23] M. B. Fasken, D. J. Morton, E. G. Kuiper, S. K. Jones, S. W. Leung, and A. H. Corbett. The RNA Exosome and Human Disease. *Methods Mol Biol*, 2062:3–33, 2020. doi: 10.1007/978-1-4939-9822-7_1.

[24] D. Wu and J. Dean. EXOSC10 sculpts the transcriptome during the growth-to-maturation transition in mouse oocytes. *Nucleic Acids Res*, 48:5349–5365, 2020. doi: 10.1093/nar/gkaa249.

[25] F. G. Petit, S. P. Jamin, P. Y. Kernanec, E. Becker, G. Halet, and M. Primig. EXOSC10/Rrp6 is essential for the eight-cell embryo/morula transition. *Dev Biol*, 483:58–65, 2022. doi: 10.1016/j.ydbio.2021.12.010.

[26] K. F. Vieux and H. J. Clarke. CNOT6 regulates a novel pattern of mRNA deadenylation during oocyte meiotic maturation. *Sci Rep*, 8:6812, 2018. doi: 10.1038/s41598-018-25187-0.

[27] H. Du, C. Chen, Y. Wang, Y. Yang, Z. Che, X. Liu, S. Meng, C. Guo, M. Xu, H. Fang, F. Wang, C. Lin, and Z. Luo. RNF219 interacts with CCR4-NOT in regulating stem cell differentiation. *J Mol Cell Biol*, 12:894–905, 2020. doi: 10.1093/jmcb/mjaa061.

[28] E. Wahle and G. S. Winkler. RNA decay machines: deadenylation by the Ccr4-not and Pan2-Pan3 complexes. *Biochim Biophys Acta*, 1829:561–70, 2013. doi: 10.1016/j.bbagrm.2013.01.003.

[29] J. A. Bohn, J. L. Van Etten, T. L. Schagat, B. M. Bowman, R. C. McEachin, P. L. Freddolino, and A. C. Goldstrohm. Identification of diverse target RNAs that are functionally regulated by human Pumilio proteins. *Nucleic Acids Res*, 46: 362–386, 2018. doi: 10.1093/nar/gkx1120.

[30] T. Bakheet, E. Hitti, and K. S. A. Khabar. ARED-Plus: an updated and expanded database of AU-rich element-containing mRNAs and pre-mRNAs. *Nucleic Acids Res*, 46:D218–D220, 2018. doi: 10.1093/nar/gkx975.

[31] R. Geissler, A. Simkin, D. Floss, R. Patel, E. A. Fogarty, J. Scheller, and A. Grim-

son. A widespread sequence-specific mRNA decay pathway mediated by hnRNPs A1 and A2/B1. *Genes Dev*, 30:1070–85, 2016. doi: 10.1101/gad.277392.116.

[32] J. M. Reyes, E. Silva, J. L. Chitwood, W. B. Schoolcraft, R. L. Krisher, and P. J. Ross. Differing molecular response of young and advanced maternal age human oocytes to IVM. *Hum Reprod*, 32:2199–2208, 2017. doi: 10.1093/humrep/dex284.

[33] Hadley Wickham. *ggplot2: Elegant Graphics for Data Analysis*. Springer-Verlag New York, 2016. ISBN 978-3-319-24277-4. URL `https://ggplot2.tidyverse.org`.

[34] Q. Q. Sha, Y. Z. Zhu, S. Li, Y. Jiang, L. Chen, X. H. Sun, L. Shen, X. H. Ou, and H. Y. Fan. Characterization of zygotic genome activation-dependent maternal mRNA clearance in mouse. *Nucleic Acids Res*, 48:879–894, 2020. doi: 10.1093/nar/gkz1111.

[35] R. Vassena, S. Boué, E. González-Roca, B. Aran, H. Auer, A. Veiga, and J. C. Izpisua Belmonte. Waves of early transcriptional activation and pluripotency program initiation during human preimplantation development. *Development*, 138:3699–709, 2011. doi: 10.1242/dev.064741.
