# Supplementary material for: Recurrent RNA edits in human preimplantation potentially enhance maternal mRNA clearance
Source: Commun Biol. 2022 Dec 21;5:1400. doi: 10.1038/s42003-022-04338-0 (PMC9772385; doi:10.1038/s42003-022-04338-0)
Supplement: Supplementary file 3 — Editorial Assessment Report [file 42003_2022_4338_MOESM3_ESM.pdf]

nature portfolio

GUIDED OPEN ACCESS

nature genetics | nature communications | communications biology

Contents of this report

1. **Manuscript details**: overview of your manuscript and the editorial team.
2. **Review synthesis**: summary of the reviewer reports provided by the editors.
3. **Editorial recommendation**: personalized evaluation and recommendation from all 3 journals.
4. **Annotated reviewer comments**: the referee reports with comments from the editors.
5. **Open research evaluation**: advice for adhering to best reproducibility practices.

About the editorial process

Because you selected the **Nature Portfolio Guided Open Access** option, your manuscript was assessed for suitability in three of our titles publishing high-quality work across the spectrum of genetics research: ***Nature Genetics***, ***Nature Communications***, and ***Communications Biology***. More information about Guided Open Access can be found here.

Collaborative editorial assessment

Your editorial team discussed the manuscript to determine its suitability for the Nature Portfolio Guided OA pilot. Our assessment of your manuscript takes into account several factors, including whether the work meets the **technical standard** of the Nature Portfolio and whether the findings are of **immediate significance** to the readership of at least one of the participating journals in the Nature Portfolio Guided Open Access genetics cluster.

Peer review

Experts were asked to evaluate the following aspects of your manuscript:

- **Novelty** in comparison to prior publications;
- **Likely audience** of researchers in terms of broad fields of study and size;
- **Potential impact** of the study on the immediate or wider research field;
- **Evidence** for the claims and whether additional experiments or analyses could feasibly strengthen the evidence;
- **Methodological detail** and whether the manuscript is reproducible as written;
- Appropriateness of the **literature review**.

Editorial evaluation of reviews

Your editorial team discussed the potential suitability of your manuscript for each of the participating journals. They then discussed the revisions necessary in order for the work to be published, keeping each journal's specific editorial criteria in mind.

Journals in the Nature portfolio will support authors wishing to transfer their reviews and (where reviewers agree) the reviewers' identities to journals outside of Springer Nature.
If you have any questions about review portability, please contact our editorial office at guidedoa@nature.com.

nature portfolio

GUIDED OPEN ACCESS

nature genetics | nature communications | communications biology

Manuscript details

Tracking number	Submission date	Decision date	Peer review type
GUIDEDOA-22-00405	Jan 26, 2022	Mar 4, 2022	Single-blind

Manuscript title

Large-scale identification of recurrent RNA edits in human embryos suggests their role in enhancing maternal mRNA clearance

Preprint: Available on ResearchSquare

Author details

Hebing Chen

Affiliation: Beijing Institute of Radiation Medicine

Editorial assessment team

**Primary editor**	**George Inglis**<br>Home journal: ***Communications Biology***<br>ORCID: **0000-0002-9069-5242**<br>Email: **george.inglis@us.nature.com**
**Other editors consulted**	**Wei Li**<br>Home journal: ***Nature Genetics***<br>ORCID: **0000-0002-7885-1775**<br><br>**Cara Eldridge**<br>Home journal: ***Nature Communications***<br>ORCID: **0000-0001-7001-2312**
**About your primary editor**	George received his PhD in Genetics and Molecular Biology from Emory University, where he studied mouse models of voltage-gated sodium channel dysfunction and epilepsy. He also has research experience in epigenomics and *in vitro* models of neuronal development. George joined the editorial team of *Communications Biology* in September 2020 and is based in the New York office.

Editorial assessment and review synthesis

**Editor's summary and assessment**	Here, the authors are interested in the role of RNA editing during embryonic development, compiling >2,000 RNA-seq samples across early human embryonic development to identify any recurrent A>I edits. They compare the distribution of these edits throughout gene bodies and families, and how they vary between abnormal embryos or maternal age, and note there are more edits in RNAs that are typically targeted for maternal clearance, suggesting that these edits might introduce miRNA binding sites (leading to degradation). Altogether, they conclude that their results provide a resource of recurrent RNA edits in human development and potential function in maternal clearance.<br><br>While the editors jointly decided to send this manuscript out to review based on the resource value of the comprehensive analysis of RNA editing across embryonic development, there were some concerns from editors at *Nature Genetics* about limited experimental validation and conceptual advance over previous studies.
**Editorial synthesis of reviewer reports**	While the reviewers agree that the topic of RNA editing in embryonic development is important, Reviewer #2 highlights the need for experimental validation of RNA editing or a role in maternal clearance, while Reviewers #1 and #3 suggest additional analyses of the compiled datasets to better report global changes in RNA editing and any relevant impacts on gene expression. The reviewers collectively stress the need for better reporting of methods and relevant literature. Taken together, these points support the initial concerns from editors and prohibit further consideration by *Nature Genetics* and *Nature Communications*.<br><br>However, *Communications Biology* would be interested in a revised manuscript that incorporates the analyses outlined by Reviewers #1 and #3, while also carefully qualifying results related to maternal clearance, and addresses discussion points raised by all three reviewers. While we would not require any of the experimental validation requested by Reviewer #2, the lack of this evidence should be explicitly discussed as a limitation of the current study.

Editorial recommendation

Nature Genetics<br><br>Revision not invited	*Nature Genetics* does not think that the degree of advance provided has matched the journal's criteria for further consideration, and thus would not invite a revision.
Nature Communications<br><br>Revision not invited	*Nature Communications* finds that the lack of experimental validation, along with the insufficient conceptual advance, do not meet our editorial bar, and prevent further consideration in the journal. We therefore cannot invite a revision.
Communications Biology<br><br>Major revisions	*Communications Biology* would be interested in a revised manuscript that incorporates the analyses outlined by Reviewers #1 and #3, while also carefully qualifying results related to maternal clearance, and addresses discussion points raised by all three reviewers. While we would not require any of the experimental validation requested by Reviewer #2, the lack of this evidence should be explicitly discussed as a limitation of the current study.

Next steps

**Editorial recommendation:**	Our top recommendation is to revise and resubmit your manuscript to *Communications Biology.* We feel the additional experiments required are reasonable to address within a 6-month time frame.
**Note**	As stated on the previous page, *Nature Genetics* and *Nature Communications* are not inviting a revision at this time. Please keep in mind that the journal will not be able to consider any appeals of their decision through Guided Open Access.

Revision

To follow our recommendation, please upload the revised manuscript files using **the link provided in the decision letter**. Should you need assistance with our manuscript tracking system, please contact Adam Lipkin, our Nature Portfolio Guided OA support specialist, at guidedOA@nature.com.

Revision checklist

- ☐ Cover letter, stating to which journal you are submitting
- ☐ Revised manuscript
- ☐ Point-by-point response to reviews
- ☐ Updated Reporting Summary and Editorial Policy Checklist
- ☐ Supplementary materials (if applicable)

Submission elsewhere

If you choose not to follow our recommendations, you can still take the reviewer reports with you.

Option 1: Transfer to another Nature Portfolio journal
Springer Nature provides authors with the ability to transfer a manuscript within the Nature Portfolio, without the author having to upload the manuscript data again. To use this service, **please follow the transfer link provided in the decision letter.** If no link was provided, please contact guidedOA@nature.com.

> *Note that any decision to opt in to In Review at the original journal is not sent to the receiving journal on transfer. You can opt in to In Review at receiving journals that support this service by choosing to modify your manuscript on transfer.*

Option 2: Portable Peer Review option for submission to a journal outside of Nature Portfolio
If you choose to submit your revised manuscript to a journal at another publisher, we can share the reviews with another journal outside of the Nature Portfolio if requested. You will need to request that the receiving journal office contacts us at guidedOA@nature.com. We have included editorial guidance below in the reviewer reports and open research evaluation to aid in revising the manuscript for publication elsewhere.

Annotated reviewer reports

The editors have included some additional comments on specific points raised by the reviewers below, to clarify requirements for publication in the recommended journal(s). However, please note that all points should be addressed in a revision, even if an editor has not specifically commented on them.

Reviewer #1 information	
**Expertise**	This reviewer has expertise in RNA editing in embryonic development.
**Editor's comments**	This reviewer acknowledges the potential interest of this topic, but emphasizes the need for better reporting of global RNA edits and raises serious concerns about the proposed maternal clearance pathway.

Reviewer #1 comments	
**Section**	**Annotated Reviewer Comments**
**Remarks to the Author: Overall significance**	In this study the authors performed integrated analysis for a large number of published human embryo RNA-seq datasets, including both normal and abnormal embryos, to systematically analyze the A-to-I editome for human early embryonic development. They proposed a regulation model in which REs enhance maternal mRNA clearance by introducing more microRNA binding sites to the 3'-untranslated regions of clearance targets. In general the biological question is of potential important and interesting. However, there are some questions that need to be addressed.
**Remarks to the Author: Strength of the claims**	1.As the author claimed they identified systematic A-to-I editome profile by analyzing the variant sites in RNA-seq. Theoretically, it is necessary to show the distribution of editing level of all variant sites. A global view is important.Also it is better to clearly describe the cutoff they used to distinguish the identified A-to-I editing sites and background noise.<br>**For the sake of reproducibility, please elaborate on all Methods. Please note that Nature Portfolio journals do not enforce a word limit on this section, and refer to the Open Research Evaluation at the end of the document for additional guidelines.**<br><br>2.In the results of Fig2A, the author only showed the overlap of editing sites in normal and other samples. However the identification of "normal" and "other" is unclear, which should be explained, at least mentioned in legends. More detailed analysis in this part would be preferred. For instance, what is the genome enrichment, gene and location (3'UTR, 5'UTR) preference for the overlap sites and the unique sites.<br>**This point would be necessary for further consideration at**

***Communications Biology*.**

3. It is better to show more detail on the indicated important ERs. For instance, the REs loss and the RNA-seq IGV trance on TTF1, which they mention in embryos with uniparental disomy and those from elder mothers. More IGVs of ERs on important genes should be present.

4.Here they used Suv39h2 as the example of RE-induced MBS-gain genes, however they did not show the editing level of this REs and the signal change of RNA-seq is also needed, as it will be a supporting evidence of the RE-induced maternal mRNA-clearance model.

5. In this manuscript the causal relation between A-I editing induced MBS and maternal RNA clearance was not logically convincing, as they only showed a week difference of MBS-gaining REs amount between 8-cell decay and undecay genes. Normally a causal relation need to be proved by the rescue experiments. More importantly the analysis of RNA level and expression pattern was barely appeared in this manuscript, which make it difficult to believe the RE-induced maternal decay model. I can understand that the batch effect and individual difference could impact the calculation of FPKM of individual genes. Here they analyzed GSE95477 which REs appeared different in yang and old mother, and therefore they can further compared the impaction of REs on RNA decay in this two group of embryos. For example for the genes loss REs in a particular stage, will the RNA abundance increased in this stage or appeared deficient decay in a later stage?

Concerns about the biological relevance of RNA editing and maternal RNA clearance prohibit further consideration by *Nature Genetics* and *Nature Communications*. Addressing this point or qualifying these conclusions would be necessary for further consideration at *Communications Biology*.

6.If the RE induced MBS enhanced the decay of maternal RNA, could the author show the time course of RE gain or loss and RNA abundance change? Also if this model worked, will the editing level of REs on maternal decay genes decreased upon the clearance of the edited transcripts. Could the RE induced MBSs related with the regulation of translation by miRNA?

This point would be necessary for further consideration at *Communications Biology*.

7. As they show REs existed from MII oocyte to 2-cell stage, however if the maternal decay occurred in 8-cell stage, why the transcripts with RE induced MBS keep stable before 8-cell stage? And how they would explain the role of RE induced MBSs in un decayed genes(Fig 6d). Here the author should explain how they identified the maternal decay genes and what is the identification of “others”

8. Could the proposed model be conservative between species? Is it possible to find conserved maternal transcripts with RE induced MBS in mice?

If feasible, please comment on this analysis, though it could also be

	**addressed as a future direction in the Discussion, for further consideration at *Communications Biology*.**
**Remarks to the Author: Reproducibility**	1. Comparison of the number of editing sites between this study and previous reports should be considered as quality control.<br><br>2. In Fig2a, only 23% sites were shared between normal and abnormal samples, what is the potential biological explanation?<br><br>3. Does the number in Fig2b represent intersect or union number of sites from different replicates of the same sample? Also, number of reproducible sites from different replicates was expected as quality control.<br><br>4. P7.l149 seems should be “median” instead of “mean”.

Reviewer #2 information

**Expertise**	This reviewer has expertise in embryonic development, maternal clearance, and epigenomics.
**Editor's comments**	While this reviewer acknowledges the potential resource value of the editome analysis, they also stress the need for experimental validation of results (particularly related to maternal clearance) and offer several useful suggestions to improve the readability and the text. These and other comments regarding the strength of novel conclusions around maternal RNA clearance prohibited further consideration by *Nature Genetics* and *Nature Communications*.

Reviewer #2 comments

Section	Annotated Reviewer Comments
**Remarks to the Author: Overall significance**	By curating and analyzing the largest human embryonic editome to date, Ding and coauthors showed that the human preimplantation embryos at various embryonic stages harbour thousands of REs that are preferably exonic and highly shared between stages at the editing site. They also proposed that these REs could potentially enhance maternal mRNA clearance, a process that is crucial for successful maternal-to-zygotic transition, by introducing more MBSs to clearance targets than to other maternal genes.
**Remarks to the Author: Impact**	This study introduced the first large-scale A-to-I RNA editome for early human embryos, the analysis of which revealed a consistent early-stage editing pattern (of REs) with probable functional importance in microRNA-based maternal mRNA clearance. The editome itself is a valuable resource for further examination of the interplay between maternal RNAs and early embryo development. However, many of the conclusions need to be experimentally confirmed. And some key hypotheses also need to be examined by bench works.<br>**While experimental validation of results would not be necessary for further consideration at *Communications Biology*, the lack of new data should be explicitly stated as a limitation and all relevant conclusions regarding maternal RNA clearance pathways should be appropriately qualified.**
**Remarks to the Author: Strength of the claims**	**Specific comments:**<br>The title of "Large-scale identification of recurrent RNA edits in human embryos" is unclear. While the authors only investigated A-to-I RNA editing in human preimplantation embryos, it is better for them to be more specific in defining the scope of research in the title.

We generally recommend that the title be written as a declarative statement (<15 words) that includes any key species, protein, or gene names.

The reader would benefit from a more detailed description of A-to-I RNA editing (i.e., frequency, sequence preference, and distribution in the 5' and 3' untranslated region, coding sequences of mRNAs) and the biochemical pathways underlying this edit (i.e., enzymes and RNA-binding proteins that mediate this post-transcriptional modification) in the Introduction section.

The authors should be sure to define abbreviations in figure legends and in the main text and minimize the number of abbreviations. As the manuscript is now, it is difficult to follow the flow on the story due to so many abbreviations.

Please avoid abbreviating terms unless they are used five or more times. We ask that you avoid all non-standard 2 letter abbreviations.

The authors showed that A-to-I RNA editing could potentially enhance maternal mRNA clearance, by introducing more MBSs to clearance targets than to other maternal genes. However, the role of an microRNA-based regulatory mechanism in maternal mRNA clearance has not been confirmed in mammalian early embryos. Therefore it is reluctant to explain the association of A-to-I RNA editing with maternal mRNA decay using this mechanism. Potential involvement of A-to-I RNA editing in PAN2-PAN3, CCR4-NOT, and exosome mediated RNA clearance pathways should be considered.

Please acknowledge these other pathways and appropriately qualify conclusions regarding maternal clearance, for further consideration at *Communications Biology*.

The occurrence and functions of A-to-I RNA editing in human preimplantation embryos are completely based on high throughput data analyses. The results of analyses are no doubt valuable, but it is equally important to experimentally confirm the location of A-to-I RNA editing on some representative maternal transcripts, and provide experimental results that support the hypotheses that A-to-I RNA editing indeed affect the dynamics of maternal mRNA clearance.

In Figure 7, there is an error about the dynamics of maternal mRNA clearance in human embryos: the major wave of maternal mRNA clearance occurs at the 8 cell stage, not after blastocyst formation as is shown in the left corner of the proposed model.

Authors need to follow the guidelines of gene symbols, protein symbols, RNA symbols. Based on our understanding gene and RNA symbols should be italicized.

Please ensure all gene and RNA symbols are italicized in the text and figures.

Reviewer #3 information	
**Expertise**	This reviewer has expertise in DNA and RNA editing methods.
**Editor's comments**	While this reviewer thinks the overarching topic is interesting, they reiterate several of Reviewer #1's concerns regarding reporting of global RNA edits and whether editing correlates with expression levels. They also highlight the need for better clarity in the methods and validation of predicted miRNA interactions using a separate tool.

Reviewer #3 comments

Section	Annotated Reviewer Comments
**Remarks to the Author: Overall significance**	In the submitted manuscript, the authors reported their analysis of A-to-I RNA editing in human embryos. They found that editing is diminished in abnormal embryos and that it can introduce microRNA binding sites in 3'UTR of maternal transcripts to facilitate their clearance. The topic of the study is interesting and might be clinically relevant to reproduction and fertility. Nevertheless, I think the work can be further improved.
**Remarks to the Author: Impact**	*Communications Biology* would be most appropriate for the manuscript.
**Remarks to the Author: Strength of the claims**	**Major comments:**<br>1) The authors should elaborate more about their analysis steps in the main text. Details of any additional filters applied (besides SNP removal) are missing from Figure 1b. As written, there seems to be nothing special about the authors' computational pipeline.<br>**A similar point was raised by Reviewer #1.**<br><br>2) Figure 1 should include the distribution of all 12 possible types of nucleotide changes (A-to-C, A-to-G, A-to-T etc) after variant calling with or without application of additional filters.<br>**A similar point was raised by Reviewer #1.**<br><br>3) Figure 1D: How was the false positive (FP) rate calculated? Please describe in main text.<br><br>4) The A-to-G percentage should be given for non-Alu and Alu sites separately.<br><br>5) The authors should calculate the Alu editing index (AEI) for each sample. Does the extent of editing correlate with ADAR expression levels?

This point would be necessary for further consideration at *Communications Biology*.

6) Figure 1 & 2 can be combined.

7) What is the number of samples for each developmental stage (Figure 3b)?

8) Figure 3c,d: It would be useful to show similar graphs for later developmental stages (morula and afterwards) as well. Is the preponderance of sites in 3'UTR observed only in the earlier developmental stages?

This point would be necessary for further consideration at *Communications Biology*.

9) Genes with recurrent edits (REs) in early stages - do their expression levels go down in later developmental stages (morula and afterwards), indicative of transcript degradation?

10) The authors wrote that "An initial scan revealed 107 edits on 76 genes that were REs in normal embryos, but completely lost in the same stage in pathological embryos and embryos from elder mothers." What is the initial scan? Please provide more details on how the 107 edits on 76 genes were shortlisted. For each site, was there sufficient sequencing coverage in the abnormal embryos? What were the editing levels of these 107 sites in the normal embryos?

11) Figure 5a,c: What are AG embryos and PG embryos? Also, what is "BI"? Please explain in the figure legend.

12) I see "amanitin" in Figure 5c. Amanitins are compounds that block RNA polymerase II, thereby inhibiting transcription. How is this drug treatment relevant to the manuscript?

13) Figure 5c: Data from GSE133854 don't look convincing. This seems to invalidate the authors' claim that there are "fewer RE-matching edits in protein-coding genes of abnormal embryos and embryos from elder mothers".

This point would be necessary for further consideration at *Communications Biology*.

14) MicroRNA prediction programs are often inaccurate. Instead of relying solely on TargetScan, can the authors use at least two different software and take the intersection?

Please validate any miRNA predictions on at least one other tool, for further consideration at *Communications Biology*.

15) Figure 6d: Where did the authors obtain the genes that "decay at 8-cell" from? I don't see any gene expression analysis. (Similar to comment #9 above.)

	**Minor comments:**<br>16) In the introduction, the authors wrote "The successful development of human embryos is based on a well-regulated network that spans multiple omic layers." It is unclear what "multiple omic layers" mean.<br><br>17) Figure 3a is redundant.<br><br>18) At the end of the results section, the authors wrote that "This hypothesis was immediately validated by the observation that ..." I suggest replacing "immediately validated" with "supported". The authors did not perform any independent experimental validations.<br><br>19) In the discussion, the authors wrote that "The role of A-to-I RNA editing in human has been ambiguous for a long time." This is not true. Multiple papers have clarified the diverse roles of editing in human.<br><br>20) In the methods section, the authors wrote "For single-cell RNA-Seq datasets, we required that the sequencing technology not be based on cell barcoding." Can the authors explain why?<br><br>21) One of the supplementary datasets (the largest one) appears to be filled with nonsensical characters.<br>**Please provide a new version of Supp Table 6; it appears to be corrupted to editors as well.**<br><br>22) There are scattered English language errors. Please proofread.<br>**Please carefully proofread the manuscript for clarity and grammar. If you would like the assistance of paid editing services to do this, we can recommend our affiliates, Nature Research Editing Service and American Journal Experts. However, please note that use of an editing service is neither a requirement nor a guarantee of publication. Free assistance is available from our resources page.**
**Remarks to the Author: Reproducibility**	Please see comments under "Strength of the claims", for example, comments #13 and #14.

Open research evaluation

Guidelines for Transparency and Openness Promotion (TOP) in Journal Policies and Practices (“TOP Guidelines”)

The recommendations and requests in the table below are aimed at bringing your manuscript in line with common community standards as exemplified by the TOP Guidelines. While every publisher and journal will implement these guidelines differently, the recommendations below are all consistent with the policies at Nature Portfolio. In most cases, these will align with TOP Guidelines Level 2.

FAIR Principles

The goal of the recommendations in the table below related to **data or code** availability is to promote the FAIR Guiding Principles for scientific data management and stewardship (*Scientific Data* **3**: 160018, 2016). The FAIR Principles are a set of guidelines for improving 4 important aspects of digital research objects: **F**indability, **A**ccessibility, **I**nteroperability and **R**eusability.

ORCID

ORCID is a non-profit organization that provides researchers with a unique digital identifier. These identifiers can be used by editors, funding agencies, publishers, and institutions to reliably identify individuals in the same way that ISBNs and DOIs identify books and articles. Thus the risk of confusing your identity with another researcher with the same name is eliminated. **The ORCID website** provides researchers with a page where your comprehensive research activity can be stored.

Springer Nature collaborates with the ORCID organization to ensure that your research contributions (as authors and peer reviewers) are correctly attributed to you. Learn more at https://www.springernature.com/gp/researchers/orcid

Data availability
**Data Availability Statement**
Thank you for including a Data Availability statement. While you have included some important information, the editors have noted that some details appear to be missing.The Data Availability Statement should be as detailed as possible and include accession codes or other unique IDs for deposited data, information about where source data can be found, and specify any restrictions to data access that may apply. At a minimum, the statement should indicate that data are available upon request and explain how data access can be granted. If data access is not possible, the reasons for this must be made clear in the Data Availability Statement.<br><br>More information about the Nature Portfolio data availability policy can be found here:<br><br>More information about formatting Data Availability Statements can be found here:<br><br>**Please clarify in the Data Availability statement where source data is located for Fig 1d, 2b-d, 3b-e, 4b-c, 5a-c, 5e, 6b-d.**
**Other data requests**
In line with community standards regarding open research, Springer Nature strongly supports data sharing and believes that all datasets on which the conclusions of the paper rely should be available to readers. We encourage authors to ensure that their datasets are either deposited in publicly available repositories (where available and appropriate) or presented in the main manuscript or additional supporting files whenever possible.<br><br>To learn more about data sharing and recommended data repositories, please see https://www.springernature.com/gp/authors/research-data-policy/repositories/12327124
All source data underlying the graphs and charts presented in the main figures must be made available as Supplementary Data (in Excel or text format) or via a generalist repository (eg, Figshare or Dryad). This is mandatory for publication in a Nature Portfolio journal, but is also best practice for publication in any venue.<br><br>**The following figures require associated source data: Fig 1d, 2b-d, 3b-e, 4b-c, 5a-c, 5e, 6b-d.**

Data citation
Please cite (within the main reference list) any datasets stored in external repositories that are mentioned within their manuscript. For previously published datasets, we ask that you cite both the related research article(s) and the datasets themselves. For more information on how to cite datasets in submitted manuscripts, please see our data availability statements and data citations policy.

Citing and referencing data in publications supports reproducible research, by increasing the transparency and provenance tracking of data generated or analysed during research. Citing data formally in reference lists also helps facilitate the tracking of data reuse and may help assign credit for individuals' contributions to research. A number of Springer Nature imprints are signatories of the Joint Declaration on Data Citation Principles, which stress the importance of data resources in scientific communication.
Thank you for depositing your dataset in a public repository. In addition to providing the link within the Data Availability statement, we ask that you also cite the dataset in the main reference list.<br><br>Citing and referencing data in publications supports reproducible research, by increasing the transparency and provenance tracking of data generated or analysed during research. Citing data formally in reference lists also helps facilitate the tracking of data reuse and may help assign credit for individuals' contributions to research. A number of Springer Nature imprints are signatories of the Joint Declaration on Data Citation Principles, which stress the importance of data resources in scientific communication.
**Code availability and citation**
Thank you for making your custom code available via Github. Upon publication, Nature Portfolio journals consider it best practice to release custom computer code in a way that allows readers to repeat the published results. Code should be deposited in a DOI-minting repository such as Zenodo, Gigantum or Code Ocean and cited in the reference list following the guidelines described in our policy pages (see link below). Authors are encouraged to manage subsequent code versions and to use a license approved by the open source initiative.<br><br>See here for more information about our code availability policies:
**Ethics**
Please ensure your Competing Interests statement includes information about all authors.<br><br>See the Nature Portfolio competing interests policy for further information: https://www.nature.com/nature-research/editorial-policies/competing-interests<br><br>The Springer Nature policy can be found here:
We believe that research that involves the use of clinical, biomedical or biometric data from human participants must only be carried out with the explicit consent of those whose data are involved. Consent must be obtained without any form of coercion and with participants' explicit understanding of the purpose for which their data will be used.<br><br>Because your study includes human participants, confirmation that all relevant ethical regulations were followed is needed for publication in any Springer Nature journal, and that informed consent was obtained. This must be stated in the Methods section, including the name of the board and institution that approved the study protocol.

Further details about the Nature Portfolio policy can be found at https://www.nature.com/commsbio/editorial-policies/ethics-and-biosecurity

Materials availability

Oligo sequences, concentrations of antibodies, and sources of cell lines must be included in the Methods (these can also be provided in a main Table and cited in the Methods). Please see the Nature Portfolio policy page for further details: https://www.nature.com/commsbio/editorial-policies/reporting-standards#availability-of-materials

Statistical reporting

Wherever statistics have been derived (e.g. error bars, box plots, statistical significance) figure legends should provide and define the n number (i.e. the sample size used to derive statistics) as a precise value (not a range), using the wording "n=X biologically independent samples/animals/cells/independent experiments/n= X cells examined over Y independent experiments" etc. as applicable. The figure legends must also indicate the statistical test used. Where appropriate, please indicate in the figure legends whether the statistical tests were one-sided or two-sided and whether adjustments were made for multiple comparisons. For null hypothesis testing, please indicate the test statistic (e.g. F, t, r) with confidence intervals, effect sizes, degrees of freedom and P values noted.

All error bars need to be defined in the figure legends (e.g. SD, SEM) together with a measure of centre (e.g. mean, median). For example, the legends should state something along the lines of "Data are presented as mean values +/- SEM" as appropriate. All box plots need to be defined in the legends in terms of minima, maxima, centre, bounds of box and whiskers and percentile.

For examples of expected description of statistics in figure legends, please see the following: https://www.nature.com/articles/s41467-019-11636-5 or https://www.nature.com/articles/s41467-019-11510-4.

When describing results as "significant" in the main text, please include details about the statistical test used and provide an exact p-value, rather than a significance threshold.

To improve reproducibility of your analyses, please provide details regarding your treatment of outliers.

To improve reproducibility of your analyses, please detail the methods used for data fitting and provide a rationale for this approach.

Data presentation

When choosing a color scheme please consider how it will display in black and white (if printed), and to users with color blindness. Please consider distinguishing data series using line patterns rather than colors, or using optimized color palettes such as those found at

https://www.nature.com/articles/nmeth.1618<br><br>The use of colored axes and labels should be avoided. Please avoid the use of red/green color contrasts, as these may be difficult to interpret for colorblind readers.
Bar graphs should only be used to present counts or proportions. If you are using bar graphs that present means/averages, it is best practice to include individual data points and/or convert the graph to a boxplot or dot-plot. You may wish to refer to this blog post about representing data distribution in plots (particularly for small datasets).
