# Supplementary material for: Recurrent RNA edits in human preimplantation potentially enhance maternal mRNA clearance
Source: Commun Biol. 2022 Dec 21;5:1400. doi: 10.1038/s42003-022-04338-0 (PMC9772385; doi:10.1038/s42003-022-04338-0)
Supplement: Supplementary file 4 — Description of Additional Supplementary Files [file 42003_2022_4338_MOESM4_ESM.docx]

File name: Supplementary Data 1

Description: Information on the embryonic datasets curated in this study.

File name: Supplementary Data 2

Description: Count of samples per developmental stage.

File name: Supplementary Data 3

Description: Mapping rates for each sample.

File name: Supplementary Data 4

Description: Mean depth across whole genome for each sample.

File name: Supplementary Data 5

Description: A-to-G proportions across all 12 nucleotide changes for each sample.

File name: Supplementary Data 6

Description: REEs lost in abnormal embryos and embryos from elder mothers.

File name: Supplementary Data 7

Description: Gene ontology enrichment of genes with REEs lost in AG embryos.

File name: Supplementary Data 8

Description: List of REEs nearly lost in embryos with uniparental disomy.

File name: Supplementary Data 9

Description: List of the union of recoding edits (including REEs and non-REEs) across all normal samples.

File name: Supplementary Data 10

Description: List of REE-targeted genes and corresponding REEs discovered in postimplantation embryos.

File name: Supplementary Data 11

Description: Details of each sample curated in this study.

File name: Supplementary Data 12

Description: Rules for the annotation of editing sites based on SnpEff results.

File name: Supplementary Data 13

Description: The source data behind Fig. 1d in the paper.

File name: Supplementary Data 14

Description: The source data behind Fig. 1f in the paper.

File name: Supplementary Data 15

Description: The source data behind Fig. 1g in the paper.

File name: Supplementary Data 16

Description: The source data behind Fig. 1i in the paper.

File name: Supplementary Data 17

Description: The source data behind Fig. 2b in the paper.

File name: Supplementary Data 18

Description: The source data behind Fig. 2c in the paper.

File name: Supplementary Data 19

Description: The source data behind Fig. 2d in the paper.

File name: Supplementary Data 20

Description: The source data behind Fig. 2e in the paper.

File name: Supplementary Data 21

Description: The source data behind Fig. 3b in the paper.

File name: Supplementary Data 22

Description: The source data behind Fig. 3c in the paper.

File name: Supplementary Data 23

Description: The source data behind Fig. 4a in the paper.

File name: Supplementary Data 24

Description: The source data behind Fig. 4b in the paper.

File name: Supplementary Data 25

Description: The source data behind Fig. 5b in the paper.

File name: Supplementary Data 26

Description: The source data behind Fig. 5c in the paper.

File name: Supplementary Data 27

Description: The source data behind Fig. 5d in the paper.

File name: Supplementary Data 28

Description: The source data behind Supplementary Figure 10 and Supplementary Figure 11 in the paper.

File name: Supplementary Data 29

Description: The sample size, difference in location (left minus right), and lower and upper bound of 95 percent confidence interval for each of the tests in Supplementary Figure 37.

File name: Supplementary Data 30

Description: The sample size, difference in location (left minus right), and lower and upper bound of 95 percent confidence interval for each of the tests in Supplementary Figure 38.

File name: Supplementary Data 31

Description: The sample size, difference in location (left minus right), and lower and upper bound of 95 percent confidence interval for each of the tests in Supplementary Figure 39.

File name: Supplementary Data 32

Description: The sample size, difference in location (left minus right), and lower and upper bound of 95 percent confidence interval for each of the tests in Supplementary Figure 41.

File name: Supplementary Data 33

Description: The sample size (spearman.n), correlation itself (spearman.cor), and lower and upper bound of 95 percent confidence interval (spearman.cor.test.95CI.LL and spearman.cor.test.95CI.UL, respectively) for each of the tests in Supplementary Figure 8. Note that "spearman.cor.to.plot" is for plotting only, where points with "spearman.cor.test.pvalue.adjusted" >= 0.05 are considered not statistically significant and moved to the NS column shown in Supplementary Figure 8.
